# Kindlin-2 enhances c-Myc translation through association with DDX3X to promote pancreatic ductal adenocarcinoma progression

**DOI:** 10.7150/thno.85421

**Published:** 2023-07-31

**Authors:** Chengmin Liu, Ke Jiang, Yanyan Ding, Aihua Yang, Renwei Cai, Panzhu Bai, Minggang Xiong, Changying Fu, Meiling Quan, Zailin Xiong, Yi Deng, Ruijun Tian, Chuanyue Wu, Ying Sun

**Affiliations:** 1 Department of System Biology, School of Life Sciences, Guangdong Provincial Key Laboratory of Cell Microenvironment and Disease Research, Shenzhen Key Laboratory of Cell Microenvironment, Southern University of Science and Technology, Shenzhen, 518055, China.; 2 Department of Human Cell Biology and Genetics, School of Medicine, Southern University of Science and Technology, Shenzhen, 518055, China.; 3 Department of Chemistry, Southern University of Science and Technology, Shenzhen, 518055, China.; 4 Department of Pathology, School of Medicine and University of Pittsburgh Cancer Institute, University of Pittsburgh, Pittsburgh, PA 15260, USA.; 5 Research Center for Chemical Biology and Omics Analysis, College of Science, Southern University of Science and Technology, Shenzhen, 518055, China.

**Keywords:** Kindlin-2, DDX3X, c-Myc, Pancreatic ductal adenocarcinoma, translation

## Abstract

**Rationale:** Pancreatic ductal adenocarcinoma (PDAC) is an aggressive solid tumor, with extremely low survival rates. Identifying key signaling pathways driving PDAC progression is crucial for the development of therapies to improve patient response rates. Kindlin-2, a multi-functional protein, is involved in numerous biological processes including cell proliferation, apoptosis and migration. However, little is known about the functions of Kindlin-2 in pancreatic cancer progression *in vivo*.

**Methods:** In this study, we employ an *in vivo* PDAC mouse model to directly investigate the role of Kindlin-2 in PDAC progression. Then, we utilized RNA-sequencing, the molecular and cellular assays to determine the molecular mechanisms by which Kindlin-2 promotes PDAC progression.

**Results**: We show that loss of Kindlin-2 markedly inhibits Kras^G12D^-driven pancreatic cancer progression *in vivo* as well as *in vitro*. Furthermore, we provide new mechanistic insight into how Kindlin-2 functions in this process, A fraction of Kindlin-2 was localized to the endoplasmic reticulum and associated with the RNA helicase DDX3X, a key regulator of mRNA translation. Loss of Kindlin-2 blocked DDX3X from binding to the 5'-untranslated region of c-Myc and inhibited DDX3X-mediated c-Myc translation, leading to reduced c-Myc-mediated glucose metabolism and tumor growth. Importantly, restoration of the expression of either the full-length Kindlin-2 or c-Myc, but not that of a DDX3X-binding-defective mutant of Kindlin-2, in Kindlin-2 deficient PDAC cells, reversed the inhibition of glycolysis and pancreatic cancer progression induced by the loss of Kindlin-2.

**Conclusion:** Our studies reveal a novel Kindlin-2-DDX3X-c-Myc signaling axis in PDAC progression and suggest that inhibition of this signaling axis may provide a promising therapeutic approach to alleviate PDAC progression.

## Introduction

Pancreatic ductal adenocarcinoma (PDAC) is a lethal malignant cancer with a 5-year survival rate of approximately 10% (in 2020), and it is projected to become the second leading cause of cancer-related death by 2030 1, 2. Efforts to understand PDAC have been greatly aided by the development of animal models of the disease. Particularly useful one is a genetically engineered mouse model, the KPC model (*LSL-Kras^G12D/+^; p53^fl/fl^; Pdx1-cre*). In these animals, Kras is hyperactivated and TP53 is deleted driven by a Cre- recombinase under the control of the pancreas-specific *Pdx1* promoter. This model is well established and has been widely used in PDAC research 3-7.

c-Myc, a pro-oncogene, is frequently overexpressed in many types of cancer, including pancreatic cancer 8. Down-regulation of c-Myc expression *in vivo* successfully inhibits cancer progression, suggesting the suppression of c-Myc could be a strategy to treat cancer patients 9. Excess c-Myc expression could be regulated in several different ways, including the regulation of c-Myc mRNA transcription, translation and protein degradation 10. Compared to transcription and protein degradation regulation, the translational control of c-Myc, especially 5' UTR-mediated translation modulation, is largely unknown.

Enhanced translational activity is a hallmark of cancer. Translation initiation, which is the initial and also limiting step of translation in eukaryotes, is often regulated by mRNA-binding chaperones. DDX3X, a DEAD-box RNA helicase family member that harbors ATPase and RNA helicase activity, plays a critical role in mRNA translation 11-13. DDX3X regulates the translation initiation of several essential genes, such as cyclin E1 14, Rac-1 15, and MITF 16. Interestingly, one common feature of these genes is a highly structured 5' UTR within their mRNAs. Recent studies defined a subset of transcriptome regulated by DDX3X, and showed that depletion of DDX3X inhibited translation of the mRNAs with complex secondary structure in their 5'UTR 17. Furthermore, dysfunction of DDX3 is involved in the progression of various cancers, including hepatocellular carcinoma 18, breast cancer 19, lung cancer 20, melanoma 21, as well as pancreatic cancer 22, suggesting investigations into the function of DDX3X might provide a novel strategy for cancer treatment.

Kindlin-2, a well-known focal adhesion protein, plays a crucial role in regulating integrin-dependent cellular events 23-29. Recent studies have reported a role for Kindlin-2 precipitates in multiple integrin-independent pathways that modulate a series of cellular functions 30-36. Kindlin-2 is essential for maintaining the hemostasis of many tissues and organs, including bone, renal glomerular, heart and smooth muscle 32, 36-39. Kindlin-2 has been reported to be overexpressed in various cancers and depletion of Kindlin-2 effectively suppressed tumor progression 33, 34, 40-42. However, to date, it is not clear whether Kindlin-2 plays a role in pancreatic cancer development* in vivo* and if so, the underlying mechanisms remain to be investigated.

In this study, we show that Kindlin-2 interacts with DDX3X, a key regulator of translation initiation, to regulate c-Myc mRNA translation. Depletion of Kindlin-2 in pancreatic cancer cells markedly decreased DDX3X-mediated c-Myc mRNA translation, reprogrammed glucose metabolism, and thereby inhibited cell proliferation *in vitro*. We also explored the function of Kindlin-2 in pancreatic cancer progression *in vivo* using a KPC mouse model. Importantly, the depletion of Kindlin-2 from pancreatic cancer cells in mice dramatically reduced c-Myc expression, inhibited tumor cell glycolysis and proliferation, and prolonged the survival of KPC mice.

## Results

### Kindlin-2 is overexpressed in human and mouse PDAC

**Figure 1:**
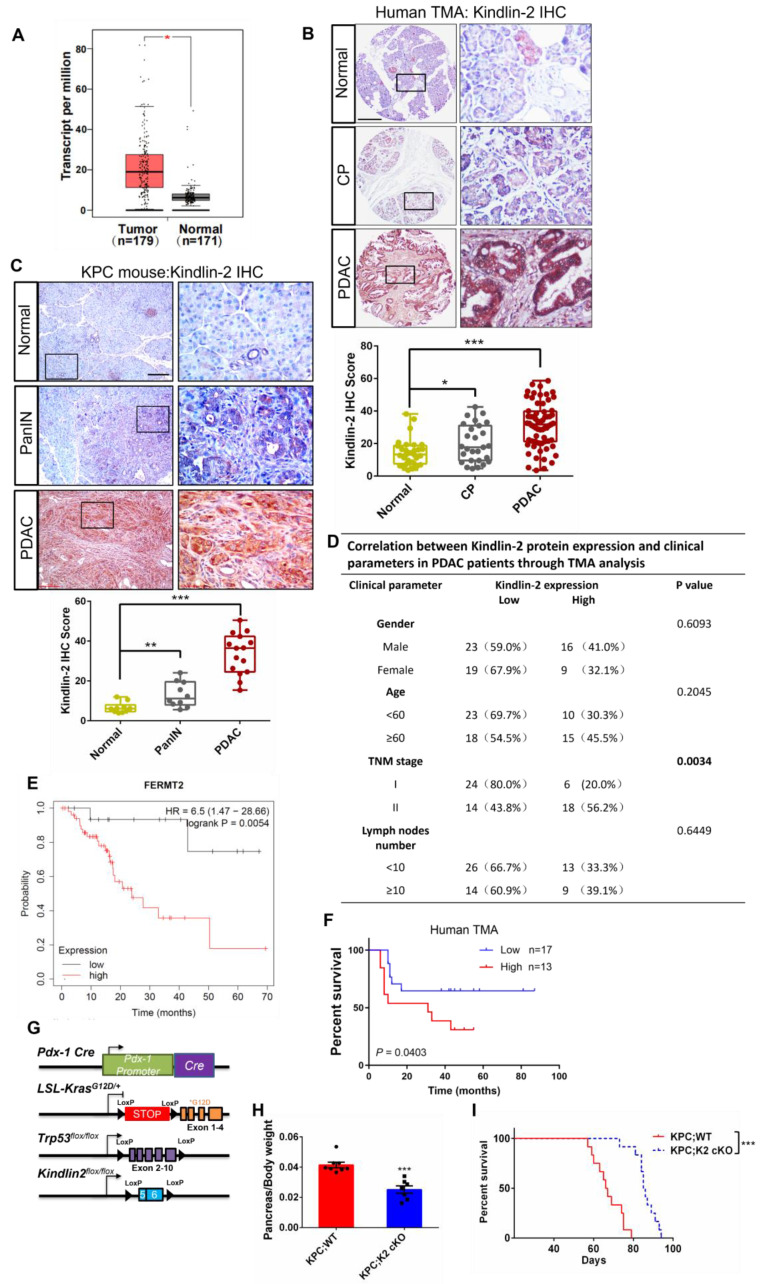
** Kindlin-2 is overexpressed in human and mouse PDAC. (A)** Analysis of *Kindlin-2* mRNA level in human PDAC (Tumor) and matched adjacent normal tissue (Normal) by GEPIA webserver (http://gepia.cancer-pku.cn/). *P < 0.05 vs. Normal. **(B)** Representative immunohistochemical staining for Kindlin-2 in TMA containing PDAC (*n* = 68) and chronic pancreatitis (CP) (*n* = 30) or normal tissues (Normal) (*n* = 33). Scale bar, 200 µm. Quantitative scoring was shown in the lower panel. *P < 0.05, ***P < 0.001 vs. Normal. (**C**) Immunohistochemical staining of Kindlin-2 in mouse normal pancreas, pancreatic intraepithelial neoplasia (PanIN) and PDAC. Scale bar, 100 µm. Quantitative scoring was shown in the lower panel. **P < 0.01, ***P < 0.001 vs. Normal. *n* = 3 mice for each group. For each mouse, the quantification was performed from at least three images. **(D)** Correlation between Kindlin-2 protein expression and clinicopathologic parameters in PDAC patients using TMA analysis. **(E)** Kaplan-Meier plot showing *Kindlin-2* (FERMT2) expression in relation to PDAC patients' disease-free survival rates. **(F)** Kaplan-Meier plot showing Kindlin-2 protein expression in relation to PDAC patients' survival rates using TMA analysis. **(G)** The diagram depicts the strategy for the generation of KPC; Kindlin-2 cKO mice (KPC; K2 cKO). **(H)** Scatter plot showing the ratio of pancreas weight to body weight of 7-week-age KPC;WT and KPC;K2 cKO mice. ***P < 0.001 vs. KPC;WT. *n* = 8 for KPC;WT; *n* = 7 for KPC;K2 cKO. **(I)** Kaplan-Meier survival analysis of KPC;WT and KPC;K2 cKO mice. ***P < 0.001 vs. KPC;WT. *n* = 12 for each group mice. TMA, tissue microarray.

To assess the clinical relevance of Kindlin-2 expression to human pancreatic cancer, we first analyzed *Kindlin-2* mRNA expression in human pancreatic cancer by using the GEPIA web server 43. The analysis showed that the mRNA level of *Kindlin-2* was up-regulated in pancreatic tumor tissues compared to non-tumor pancreatic tissues (Figure 1A). To determine the relation between Kindlin-2 protein expression and pancreatic cancer, we performed Kindlin-2 immunohistochemistry (IHC) on a human tissue microarray (TMA) containing 68 PDAC, 30 chronic pancreatitis (CP), and 33 non-tumor specimens. Concomitant with the mRNA expression analysis, Kindlin-2 protein expression was barely detectable in the non-tumor human pancreatic tissues, slightly elevated in CP tissues, and remarkably increased in PDAC samples (Figure 1B). These observations were confirmed by staining mouse pancreatic tissue samples. Consistent with human TMA data, Kindlin-2 protein expression was slightly higher in mouse pancreatic intraepithelial neoplasia (PanINs) than in normal pancreatic tissues, and progressively increased with the progression of pancreatic adenocarcinoma, suggesting Kindlin-2 might play a crucial role in regulating PDAC progression (Figure 1C). To assess the clinical significance of Kindlin-2 expression in human pancreatic cancer progression, we further analyzed the correlation of Kindlin-2 protein levels with pancreatic cancer patients' clinicopathologic parameters, including gender, age, tumor, node and metastasis (TNM) stage, and the number of lymph nodes. The results showed that Kindlin-2 expression was significantly correlated with TNM stage (*p* = 0.0034), but not with age, gender, or lymph nodes number (Figure 1D).

Next, to investigate the impact of Kindlin-2 expression on the clinical outcomes of patients with pancreatic cancer, we analyzed the prognostic value of *Kindlin-2* mRNA in human pancreatic cancer patients using Kaplan-Meier plots from the data set in Reference 44. The analysis revealed that PDAC patients with high Kindlin-2 expression had reduced rates of disease-free survival (Figure 1E). Consistently, the Kaplan-Meier analysis showed that the PDAC patients with higher Kindlin-2 protein expression had poorer prognosis as compared to patients with lower Kindlin-2 protein expression (Figure 1F). Collectively, these results strongly suggested an important role for Kindlin-2 in the pathology of pancreatic cancer.

To directly investigate the role of Kindlin-2 in regulating PDAC progression, we generated two experimental groups of mice, wild-type KPC and littermate KPC mice with pancreas-specific knockout of Kindlin-2: *LSL-Kras^G12D/+^; p53^fl/fl^; Pdx1-cre; Kindlin-2^ +/+^* (KPC;WT) and *LSL-Kras^G12D/+^; p53^fl/fl^; Pdx1-cre; Kindlin-2^ fl/fl^* (KPC;K2 cKO) (Figure 1G and Figure S1). Mice of all genotypes were viable and born at the expected Mendelian frequency. We first evaluated the body weights, global behavior, and key organ phenotypes (e.g. liver, kidney, and lungs) in KPC;K2 cKO mice. The results showed that KPC;K2 cKO mice displayed normal behaviors and normal development of a set of key organs, such as the liver, kidney, and lungs (Figure S2). The body weights of KPC;K2 cKO mice were not significantly different from those of the KPC;WT mice at 7 weeks of age (Figure S3), albeit the ratio of pancreas weight to body weight of KPC;K2 cKO mice was slightly reduced compared with KPC;WT mice at 7 weeks of age (Figure 1H). Taken together, these data indicate that pancreas-specific knockout Kindlin-2 did not significantly cause dysfunction of other key tissues and organs. We further evaluated the overall survival rate between KPC;WT and KPC;K2 cKO mice (Figure 1I). Consistent with the previous reports, our data showed that the median survival of KPC;WT was around 67 days, and all KPC;WT mice died within 79 days. Whereas KPC;K2 cKO mice exhibited a prolonged median survival of 88 days, and all KPC;K2 cKO mice died within 95 days, suggesting Kindlin-2 deletion in pancreatic tumor cells significantly prolonged survival of KPC mice (Figure 1I).

### Kindlin-2 deletion alleviates PDAC progression

**Figure 2:**
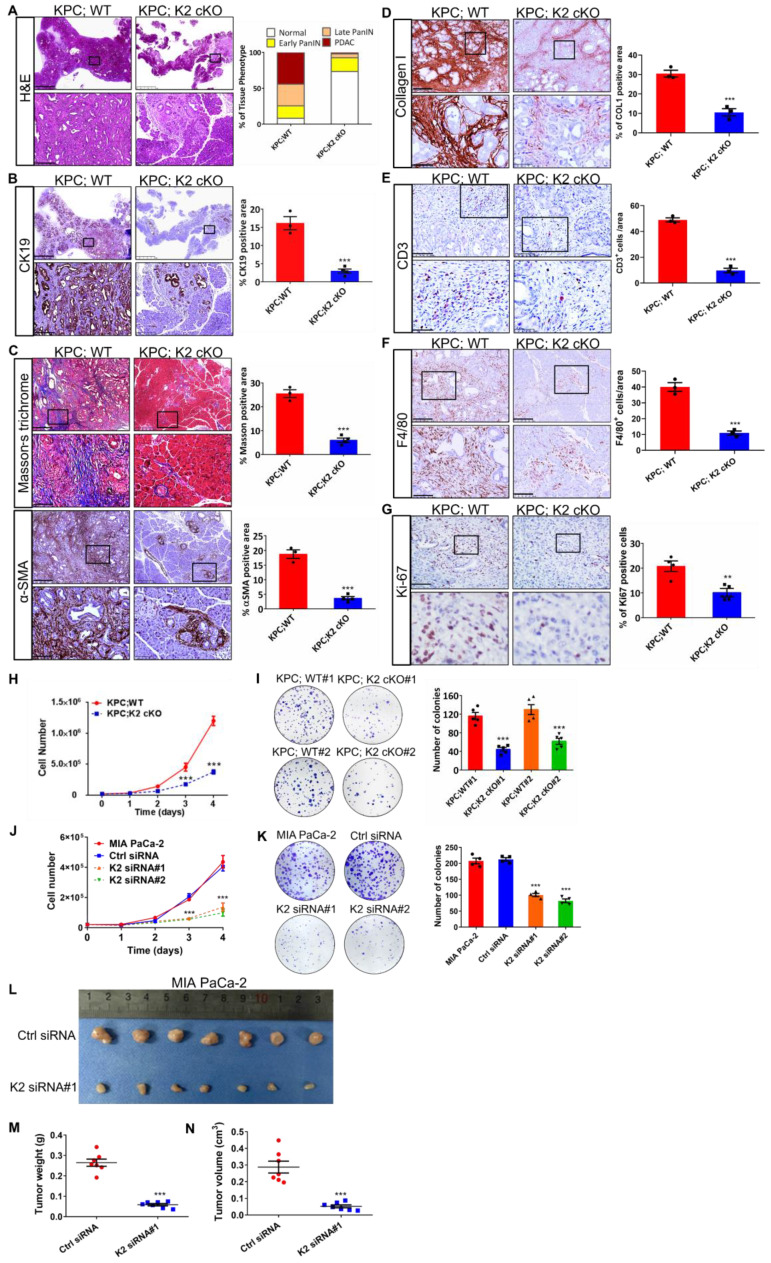
** Kindlin-2 deletion inhibits pancreatic cancer progression and reduces pancreatic tumor cell proliferation. (A)** Representative histologic images of H&E staining in the pancreatic tumors. Scale bar: 2.5 mm (upper panel); 250 µm (lower panel). Quantification analysis was shown in the right panel. *n* = 3 for KPC;WT; *n* = 4 for KPC;K2 cKO.** (B)** Representative histologic images of CK19 staining in the pancreatic tumors. Scale bar: 2.5 mm (upper panel); 250 µm (lower panel). Quantification analysis was shown in the right panel. ***P < 0.001 vs. KPC;WT. *n* = 3 for KPC;WT; *n* = 4 for KPC;K2 cKO.** (C)** Representative histologic images of Masson's trichrome staining and α-SMA staining in the pancreatic tumors. Scale bar: 500 µm (upper panel);100 µm (lower panel). Quantification analysis was shown in the right panel. ***P < 0.001 vs. KPC;WT. *n* = 3 for KPC;WT; *n* = 4 for KPC;K2 cKO.** (D)** Representative histologic images of collagen I staining in the pancreatic tumors. Scale bar: 250 µm (upper panel); 50 µm (lower panel). Quantification analysis was shown in the right panel. ***P < 0.001 vs. KPC;WT. *n* = 3 for each group.** (E)** Representative histologic images of CD3 staining in the pancreatic tumors. Scale bar: 100 µm (upper panel); 50 µm (lower panel). Quantification analysis was shown in the right panel. ***P < 0.001 vs. KPC;WT. *n* = 3 for each group.** (F)** Representative histologic images of F4/80 staining in the pancreatic tumors. Scale bar: 250 µm (upper panel); 100 µm (lower panel). Quantification analysis was shown in the right panel. ***P < 0.001 vs. KPC;WT. *n* = 3 for each group.** (G)** Representative histologic images of Ki-67 staining in the pancreatic tumors. Scale bar: 50 µm. Quantification analysis was shown in the right panel. **P < 0.01 vs. KPC;WT. *n* = 4 for KPC;WT; *n* = 4 for KPC;K2 cKO.** (H)** Knockout of Kindlin-2 in mouse primary PCCs led to a significant decrease in cell viability, as measured by cell number counting at the indicated time points. ***P < 0.001 vs. KPC;WT. *n* = 3 independent experiments.** (I)** Knockout of Kindlin-2 in mouse primary PCCs led to a significant decrease in anchorage-dependent colony-forming abilities. ***P < 0.001, vs. KPC;WT#1. *n* = 5 independent experiments.** (J)** Knockdown of Kindlin-2 in human MIA PaCa-2 cells led to a significant decrease in cell viability, as measured by cell number counting at the indicated time points. ***P < 0.001 vs. MIA PaCa-2. *n* = 3 independent experiments.** (K)** Knockdown of Kindlin-2 in human MIA PaCa-2 cells led to a significant decrease in anchorage-dependent colony-forming abilities. ***P < 0.001, vs. MIA PaCa-2. *n* = 4 independent experiments.** (L-N)** Tumor growth from the orthotopic inoculation of control or Kindlin-2 knockdown MIA PaCa-2 cells in the pancreas of nude mice. Representative images of tumors **(L)**, quantification of tumor weight **(M)** and tumor volume** (N)** at day 14 after inoculation were shown. ***P < 0.001 vs. Ctrl siRNA. *n* = 7 mice for each group.

To determine the cause of prolonged survival in KPC;K2 cKO mice, we isolated pancreatic tissues from 7-week-old KPC mice, a time point at which pancreatic tissues are occupied by both low- and high-grade pancreatic cancer 45. Histological analysis revealed a predominant increase of normal pancreatic acinar tissues in KPC;K2 cKO mice compared with KPC;WT mice (Figure 2A). Approximately 91% of pancreata were replaced by pancreatic intraepithelial neoplasia (PanIN) or PDAC in 7-week-old KPC;WT mice, whereas only 27% pancreas with altered acinar architecture in KPC;K2 cKO mice (Figure 2A). Accordingly, immunohistochemical (IHC) staining of Cytokeratin 19 (CK19), a ductal epithelial marker, also showed a dramatic reduction of CK19-positive lesions in KPC;K2 cKO mice (Figure 2B), suggesting Kindlin-2 ablation delays PDAC progression. Given the microenvironment of PDAC strongly influences tumorigenesis, we sought to examine whether depletion of Kindlin-2 could remodel the tumor microenvironment. Interestingly, the fibrotic areas measured by Masson trichrome staining and α-smooth muscle actin (α-SMA) IHC staining were alleviated in KPC;K2 cKO mice (Figure 2C). Collagen is the most abundant component of the extracellular matrix (ECM) in pancreatic cancer, and ECM increased during PDAC progression 46. IHC staining showed that collagen densities were decreased in KPC;K2 cKO compared to KPC;WT mice, indicating that the loss of Kindlin-2 reduced collagen ECM deposition in PDAC (Figure 2D). High amounts of macrophage and T cell infiltration are often found in human PDAC, which promote tumor growth by releasing pro-tumorigenic factors 47-49. Therefore, we tested the effects of Kindlin-2 on the PDAC immune microenvironment. A strong reduction of F4/80^+^ tumor-associated macrophages (TAMs) and CD3^+^ T cells infiltration in tumor sections was observed in KPC;K2 cKO compared to KPC;WT mice (Figure 2E-F), suggesting that Kindlin-2 depletion inhibited the infiltration of TAMs and T cells, and then possibly suppressed the inflammatory microenvironment in PDAC to affect tumor progression 47-49. Pancreatic cancer cells have excessive proliferation ability 50. To address whether Kindlin-2 depletion delayed PDAC progression through influencing pancreatic cancer cell proliferation rates, we performed Ki-67 staining. The results showed a significantly decreased percentage of Ki-67-positive cells in KPC;K2 cKO tumor lesions compared with KPC;WT tumors (Figure 2G). To corroborate the *in vivo* data, we performed *in vitro* cell proliferation assay. Results showed that pancreatic cancer cells lacking Kindlin-2 displayed lower proliferation rates and lower frequencies of colony formation, indicating that the loss of Kindlin-2 has a strong inhibitory effect on mouse primary PCC proliferation (Figure 2H-I). To confirm these findings in human PDAC, we knocked down Kindlin-2 in MIA PaCa-2 cells, which are widely used human-derived PDAC cells (Figure S4). Similar to what we found in primary mouse PCCs, the knockdown of Kindlin-2 in MIA PaCa-2 cells inhibited pancreatic cancer cell proliferation (Figure 2J-K). To verify this *in vitro* data, we analyzed the effects of Kindlin-2 on pancreatic cancer progression using a xenograft model by implanting human wild-type or Kindlin-2 knockdown MIA PaCa-2 cells into mice. To do this, nude mice were orthotopically injected with an equal number of MIA PaCa-2 cells expressing control siRNA or Kindlin-2 siRNA#1. Similar to what we observed in KPC; cKO mice, nude mice injected with Kindlin-2 knockdown MIA PaCa-2 cells showed less tumor burden compared to mice injected with control cells (Figure 2L-N), further confirming the pro-tumorigenic effects of Kindlin-2 on pancreatic cancer. Taken together, these results indicated that loss of Kindlin-2 inhibits PCC proliferation and delays PDAC progression.

### Kindlin-2 promotes pancreatic cancer cell proliferation through regulation of c-Myc translation process

**Figure 3:**
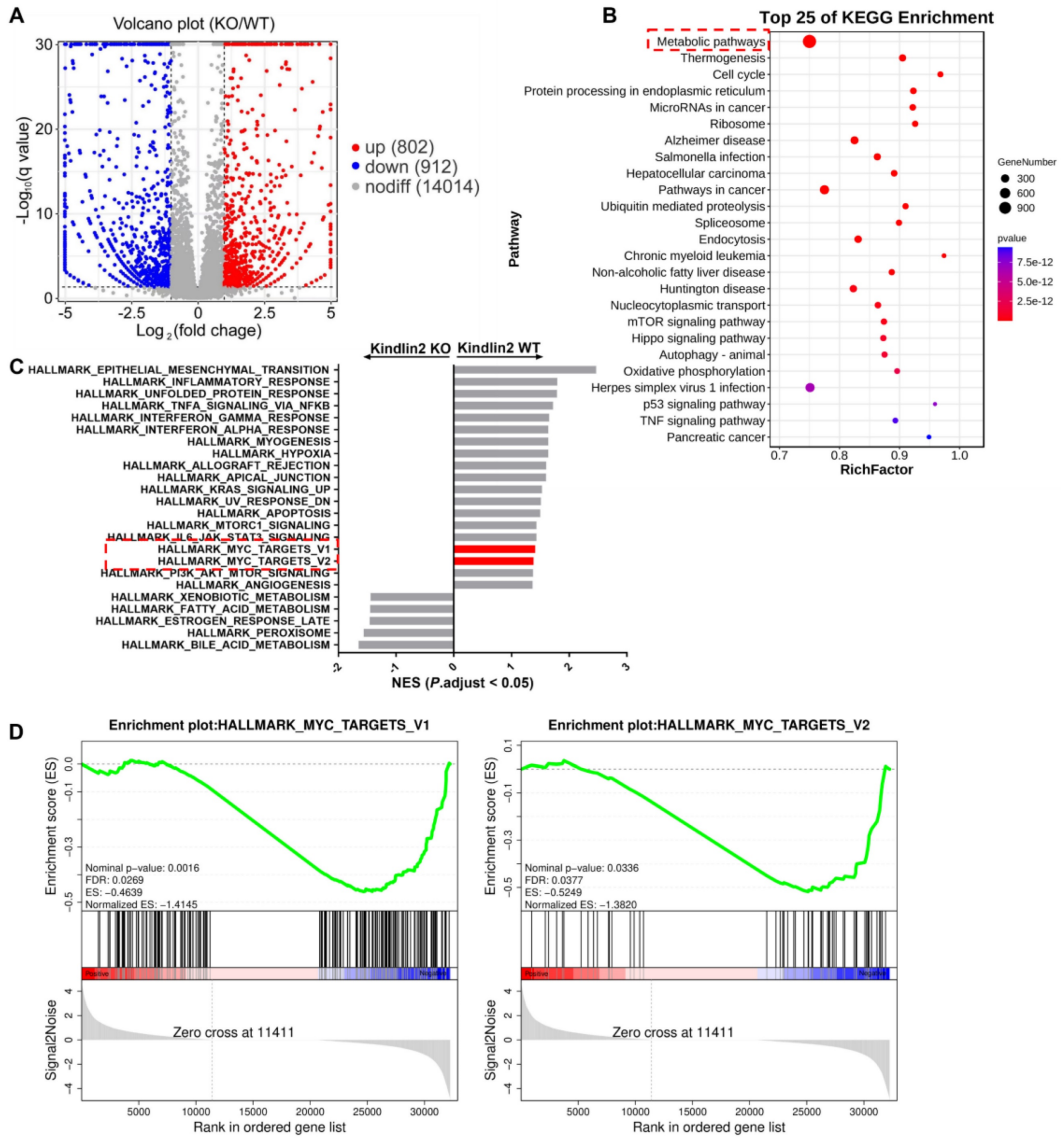
** RNA-seq analysis of primary PCCs isolated from KPC;WT and KPC; K2 cKO mice. (A)** Volcano plot of gene expression (KPC;K2 cKO vs. KPC;WT mice).** (B)** KEGG pathway enrichment analysis of differentially expressed genes in primary PCCs from KPC;K2 cKO and KPC;WT mice. **(C)** GSEA analysis of enriched gene sets in the comparison of primary PCCs from KPC;K2 cKO vs. KPC;WT mice.** (D)** GSEA analysis showing c-Myc targets-V1 and targets-V2 gene sets enriched in KPC;WT PCCs. KEGG, Kyoto Encyclopedia of Genes and Genomes; GSEA, Gene Set Enrichment Analysis.

**Figure 4:**
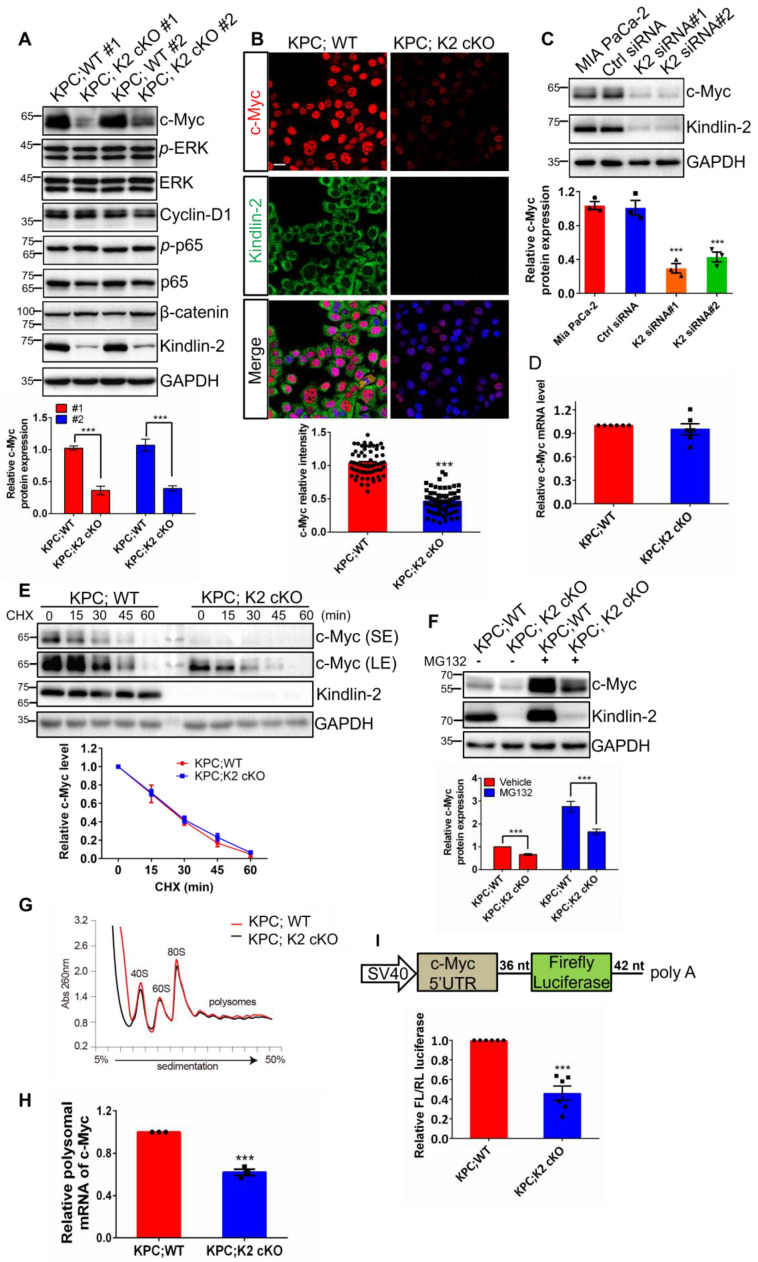
** Kindlin-2 promotes pancreatic cancer cell proliferation through regulation of the translation of c-Myc. (A)** Immunoblotting analysis of a subset of protein expression (as specified in the figure) in isolated primary PCCs; Quantification analysis was shown in the lower panel. ***P < 0.001 vs. KPC;WT. *n* = 3 independent experiments.** (B)** Representative images of immunofluorescence staining for Kindlin-2 (green) and c-Myc (red) in mouse primary PCCs. Scale bar: 20 µm. Quantification analysis was shown in the lower panel. At least 15 images in each group were analyzed. ***P < 0.001 vs. KPC;WT. *n* = 4 independent experiments.** (C)** Immunoblotting analysis of c-Myc protein expression in control (Ctrl siRNA) and Kindlin-2 knockdown (K2 siRNA#1 and K2 siRNA#2) MIA PaCa-2 cells. Quantification analysis was shown in the lower panel. ***P < 0.001 vs. MIA PaCa-2. *n* = 3 independent experiments.** (D)** qPCR analysis of *c-Myc* mRNA expression in PCCs. *n* = 6 independent experiments.** (E)** Immunoblotting analysis of c-Myc protein level in PCCs treated with cycloheximide (CHX) for different time points as indicated (upper panel). Quantification analysis was shown in the lower panel. *n* = 4 independent experiments. SE, short exposure; LE, long exposure.** (F)** Immunoblotting analysis of c-Myc protein level in PCCs treated with proteasomal inhibitor MG132 for 6 h (upper panel). Vehicle, DMSO; MG132, 10 µM. Quantification analysis was shown in the lower panel. ***P < 0.001 vs. KPC;WT. *n* = 5 independent experiments.** (G)** Representative polysome profiles from PCCs. Absorbance (Abs) at 260 nm was shown as a function of sedimentation. **(H)** The fractions of polysomes were mixed together and the RNA of the mixture was isolated and subjected to qPCR analysis to determine the polysomal mRNA level of c-Myc. ***P < 0.001 vs. KPC;WT. *n* = 3 independent experiments.** (I)** Upper panel: schematic of c-Myc 5'UTR-mediated translation (*Firefly* luciferase as a reporter gene). Lower panel: luciferase assay of c-Myc 5'UTR-mediated translational activity in primary PCCs. ***P < 0.001 vs. KPC;WT. *n* = 6 independent experiments. FL, *firefly*; RL, *Renilla*.

To elucidate the potential mechanisms by which Kindlin-2 promotes pancreatic cancer cell proliferation, we performed RNA sequencing (RNA-seq) analysis with primary wild-type and Kindlin-2 knockout PCCs to comprehensively understand the signaling pathways through which Kindlin-2 regulated pancreatic cancer cell proliferation. In these experiments, 912 genes were down-regulated and 802 genes were up-regulated in the Kindlin-2 knockout PCCs compared to wild-type PCCs (Figure 3A). Consistent with other findings described in this paper, gene ontology enrichment analysis using genes from the Kyoto Encyclopedia of Genes and Genomes (KEGG) revealed that the most down-regulated gene clusters in Kindlin-2 knockout PCCs were related to tumor metabolic pathways (Figure 3B). Furthermore, gene set enrichment analysis (GSEA) indicated that 17 gene sets were significantly down-regulated in Kindlin-2 knockout PCCs, including inflammatory response, hypoxia, mTORC1, c-Myc, and other signaling pathways (Figure 3C-D). Given the crucial role of c-Myc in the regulation of tumor metabolic pathways and pancreatic cancer progression, we sought to investigate whether Kindlin-2 regulates c-Myc-mediated pancreatic cancer progression. To do this, we explored the effects of Kindlin-2 on c-Myc expression. Consistent with RNA-seq data, knockout of Kindlin-2 in mouse primary PCCs markedly reduced c-Myc expression level, whereas other key signaling molecules involved in the regulation of cell proliferation including p-ERK/ERK, p-p65/p65, cyclin D1, and β-catenin were not significantly altered (Figure 4A). Kindlin-2-deficiency induced down-regulation of c-Myc expression was further confirmed by immunofluorescence staining of mouse PCCs (Figure 4B). We also repeated the effect of reduced Kindlin-2 on c-Myc expression by using human MIA PaCa-2 cells and obtained similar results (Figure 4C). c-Myc protein regulation has been reported to be achieved by inhibiting mRNA transcription, suppressing translation or promoting protein degradation 51-53. To illustrate which process of c-Myc was regulated by Kindlin-2, we first compared the mRNA level of c-Myc between wild-type and Kindlin-2 knockout PCCs. The results showed that the mRNA level of c-Myc was not significantly changed by the depletion of Kindlin-2 (Figure 4D). Next, to address whether Kindlin-2 affects c-Myc protein degradation, cycloheximide (CHX) pulse- chase assays were performed. As shown in Figure 4E, Kindlin-2 ablation failed to alter the median half-life of c-Myc upon inhibition of protein synthesis (Figure 4E). Given previous reports they implied c-Myc protein undergoes proteasome-mediated degradation 53, we validated the CHX chase analysis by treating cells with proteasomal inhibitor MG132. As expected, MG132 could not reverse Kindlin-2 deficiency-induced down-regulation of c-Myc, indicating Kindlin-2 is unlikely to regulate c-Myc levels through protein degradation (Figure 4F). Finally, we asked whether Kindlin-2 affects the c-Myc translation process. To answer this question, we performed sucrose gradient sedimentation to analyze the distribution of total RNA on polyribosomes in primary PCCs isolated from KPC;K2 cKO and KPC;WT mice (Figure 4G). Interestingly, we found that loss of Kindlin-2 did not significantly modulate the global ribosome profiles, suggesting Kindlin-2 depletion did not lead to a global translation inhibition. To delineate the role of Kindlin-2 on specific c-Myc gene translation, we further analyzed c-Myc transcript from polysomal fractions prepared from PCCs from KPC;K2 cKO and KPC;WT mice.

c-Myc polysomal mRNA levels were drastically reduced in Kindlin-2 deficient PCCs, supporting the view that Kindlin-2 selectively modulates c-Myc mRNA translation (Figure 4H). 5'- and 3'- untranslated regions (UTRs) play critical roles in the control of mRNA translation^54^-^56^. To determine if Kindlin-2 regulates c-Myc expression through 5'- or 3'- UTR-mediated translation, we constructed a *firefly* luciferase reporter containing the 581 nt 5'UTR or 453 nt 3'UTR of mouse c-Myc and co-transfected this reporter with a *Renilla* luciferase, an internal control reporter plasmid, into PCCs from KPC;K2 cKO and KPC;WT mice. After normalizing with the control *Renilla* luciferase, we found that loss of Kindlin-2 remarkably impaired c-Myc 5'-UTR mediated reporter translation efficiency (Figure 4I). In contrast, Kindlin-2 ablation failed to affect c-Myc 3'UTR-mediated translation efficiency (Figure S5), implying Kindlin-2 stabilizes c-Myc expression primarily through regulation of 5'UTR-mediated translation. Taken together, these results indicate that Kindlin-2 controls c-Myc expression through regulation of c-Myc mRNA translation, but not transcription or degradation processes.

### Kindlin-2 associates with DDX3X

**Figure 5:**
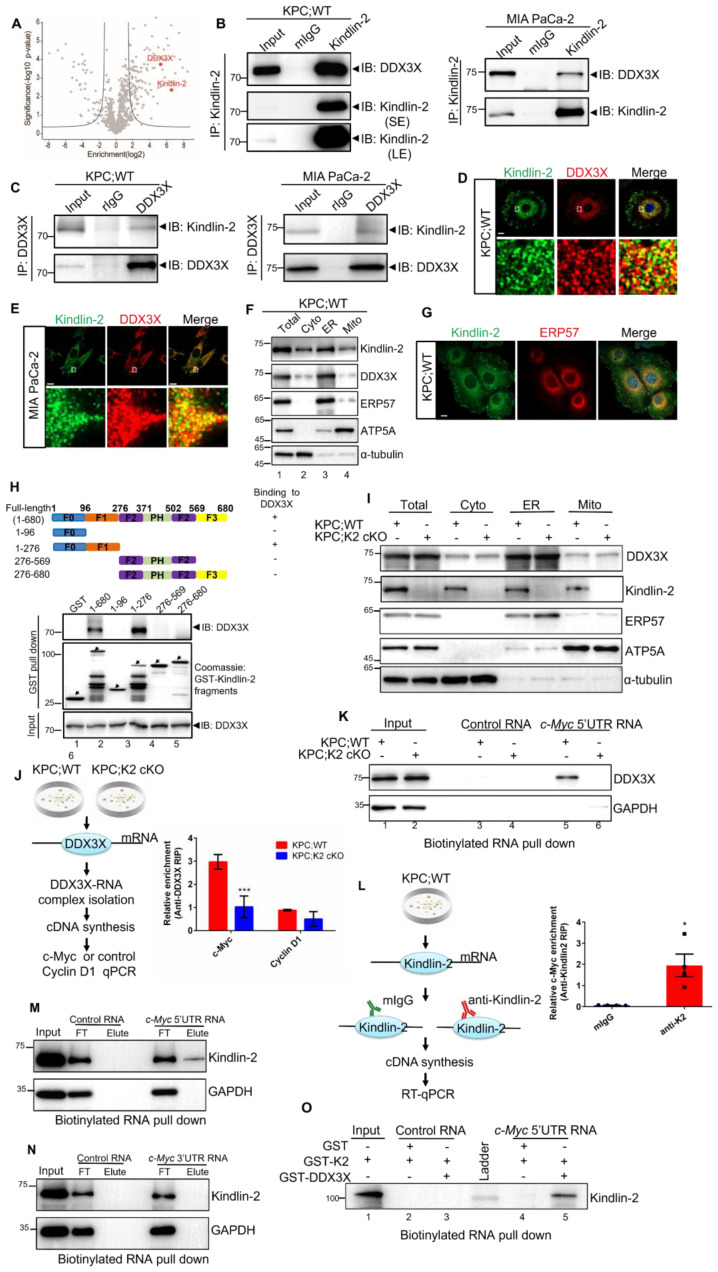
** Kindlin-2 associates with DDX3X and regulates DDX3X binding to c-Myc mRNA. (A)**Volcano plot showing Kindlin-2 interacting proteins identified using Kindlin-2 immunoprecipitation followed by Mass Spectrometry (MS) in PCCs isolated from KPC;WT mice. The positions of Kindlin-2 and DDX3X are indicated. **(B)** Primary mouse PCCs (left panel) or Human MIA PaCa-2 (right panel) cell lysates were immunoprecipitated with anti-Kindlin-2 antibody or mouse control IgG (mIgG) followed by immunoblotting with antibodies as indicated. SE, short exposure; LE, long exposure.** (C)** Primary mouse PCCs (left panel) or Human MIA PaCa-2 (right panel) cell lysates were immunoprecipitated with anti-DDX3X antibody or rabbit control IgG (rIgG) followed by immunoblotting with antibodies as indicated. **(D and E)** Primary PCCs isolated from KPC;WT mice** (D)** or Human MIA PaCa-2 cells** (E)** were co-stained with mouse anti-Kindlin-2 and rabbit anti-DDX3X antibodies. Scale bar: 10 µm.** (F)** The cytosolic fraction (Cyto, lane 2), endoplasmic reticulum (ER) fraction (ER, lane 3), mitochondrial fraction (Mito, lane 4) and total cell lysates (Total, lane 1) from PCCs isolated from KPC;WT were analyzed by immunoblotting with antibodies as indicated.** (G)** Primary PCCs isolated from KPC;WT were co-stained with anti-Kindlin-2 and anti-ERP57 (ER marker) antibodies. Scale bar: 10 µm.** (H)** Mapping the subdomains of Kindlin-2 that mediated the association with DDX3X. Upper panel: schematic illustration of various Kindlin-2 fragments that were used in the GST pull-down assay. Lower panel: GST-fusion proteins containing various fragments of Kindlin-2 were used to pull-down endogenous DDX3X from PCCs isolated from KPC;WT mice.** (I)** The cytosolic fraction, ER fraction, mitochondrial fraction and total cell lysates from PCCs were analyzed by immunoblotting with antibodies as indicated.** (J)** Left panel: RNA-immunoprecipitation (RIP) strategy used to investigate DDX3X binding to c-Myc mRNA in PCCs. Right panel: c-Myc mRNA, but not cyclin D1 mRNA, was significantly enriched in DDX3X immunoprecipitated from PCCs isolated from KPC;WT compared to that from KPC;K2 cKO littermates. ***P < 0.001 vs. KPC;WT. *n* = 3 independent experiments.** (K)** Pull-down assay using the biotinylated c-Myc 5'-UTR RNA with cell lysates of mouse primary PCCs, followed by immunoblotting analysis with antibodies as indicated. **(L)** Left panel: RNA-immunoprecipitation (RIP) strategy used to investigate Kindlin-2 binding to c-Myc mRNA in PCCs. Right panel: c-Myc mRNA was significantly enriched in Kindlin-2 immunoprecipitants, but not in control IgG (mIgG) immunoprecipitants. *P < 0.05 vs. mIgG. *n* = 4 independent experiments.** (M)** Pull-down assay using the biotinylated c-Myc 5' UTR RNA or control RNA with cell lysates of mouse primary PCCs, followed by immunoblotting analysis with antibodies as indicated. FT, flow through.** (N)** Pull-down assay using the biotinylated c-Myc 3' UTR RNA or control RNA with cell lysates of mouse primary PCCs, followed by immunoblotting analysis with antibodies as indicated. FT, flow through.** (O)** Pull-down assay using the biotinylated c-Myc 5'UTR RNA or control RNA with purified GST-Kindlin-2 (GST-K2)/GST or GST-K2/GST-DDX3X, followed by immunoblotting analysis with anti-Kindlin-2 antibodies.

To explore the mechanism underlying the regulation of c-Myc mRNA translation by Kindlin-2, we attempted to identify Kindlin-2-associated proteins that participate in the translation initiation process by Nanoscale liquid chromatography coupled to tandem mass spectrometry (nano LC-MC/MS) approach 32 (Figure 5A). Among the potential Kindlin-2-interacting candidates, DDX3X was chosen for further investigation because DDX3X, an RNA helicase protein, is known to function as a translational regulator to facilitate the translation initiation of several essential genes 13-17. Kindlin-2-DDX3X association was first verified by sequential immunoprecipitation (IP) experiments with either anti-Kindlin-2 or anti-DDX3X antibodies using mouse-isolated PCCs or human MIA PaCa-2 cells. The results validated that Kindlin-2 is associated with DDX3X (Figure 5B-C). To further examine the location where the Kindlin-2 and DDX3X interacted, we performed immunofluorescence co-staining of Kindlin-2 and DDX3X in mouse primary PCCs or human MIA PaCa-2 cells. Interestingly, DDX3X was not detected in the focal adhesions where a fraction of Kindlin-2 was localized. However, DDX3X did co-localize with Kindlin-2 in peri-nuclear regions (Figure 5D-E). DDX3X has been previously reported to distribute at the endoplasmic reticulum (ER) regions to mediate ER-associated protein translation 57. To investigate whether Kindlin-2 co-localized with DDX3X at ER region, we isolated ER, mitochondrial and other cytosolic fractions from mouse primary PCCs. ATP5A (a mitochondrial marker), ERP57 (an ER marker) and α-tubulin (a cytosolic marker) were used to indicate different cellular fractions. Consistent with the co-staining data, subcellular fractionation analysis revealed that the majority of Kindlin-2 and DDX3X were detected in the ER fractions, albeit a small amount of Kindlin-2 and DDX3X were also detected in the mitochondrial and cytosolic fraction (Figure 5F), indicating Kindlin-2 mainly associated with DDX3X in the ER. To further test the Kindlin-2 localization in ER, we performed co-staining of Kindlin-2 and ERP57 (an ER marker). The results confirmed our hypothesis (Figure 5G). Finally, to identify which subdomain of Kindlin-2 binds to DDX3X, we purified glutathione S-transferase (GST)-fusion proteins containing various Kindlin-2 fragments and tested their binding abilities with DDX3X. As shown in Figure 5H, the full-length and F0F1 subdomains of Kindlin-2 (lanes 2 and 4), but not F0, F2 or F2F3 subdomains (lanes 3, 5 and 6), could pull down DDX3X, suggesting that Kindlin-2 binds to DDX3X through its F1 subdomain (Figure 5H).

### Kindlin-2 deletion inhibits DDX3X-mediated c-Myc translation

DDX3X has been previously reported to facilitate translation initiation of RNAs that contain long and/or secondary structured 5'UTR 12, 17. RNA fold analysis revealed that the c-Myc 5'UTR contains a long and stable secondary structure with an estimated free energy of -257.50 kal/mol (Figure S6). Therefore, we hypothesized that Kindlin-2 might regulate the c-Myc translation process through a Kindlin-2-DDX3X axis. To test this hypothesis, we first explored the impact of Kindlin-2 on DDX3X protein expression. To our surprise, the loss of Kindlin-2 did not change DDX3X total protein expression (Figure S7). We then evaluated whether Kindlin-2 depletion altered DDX3X ER subcellular localization. Results indicated that the deficiency of Kindlin-2 did not cause the relocation of DDX3X from the ER fraction to other cellular fractions, suggesting that Kindlin-2 is not necessary to regulate the cellular localization of DDX3X (Figure 5I). We next asked whether Kindlin-2 affects DDX3X's binding to c-Myc mRNA. To test this, we examined DDX3X-c-Myc mRNA binding abilities in PCCs from KPC;K2 cKO and KPC;WT mice using RNA immunoprecipitation (RIP) followed by qRT-PCR (Figure 5J). Strikingly, significantly less c-Myc mRNA was co-immunoprecipitated with DDX3X in Kindlin-2 knockout PCCs compared to control cells. As a control, cyclin D1 (DDX3X-insensitive gene) 14 mRNA showed no difference in DDX3X RIP samples from wild-type or Kindlin-2 knockout PCCs (Figure 5J). To further verify these data, we performed an RNA affinity pull-down assay using biotinylated c-Myc 5' UTR or c-Myc 3'UTR. The results showed that depletion of Kindlin-2 diminished the association of the DDX3X proteins with c-Myc 5' UTR (Figure 5K). In contrast, c-Myc 3'UTR, unlike c-Myc 5'UTR, did not associate with DDX3X in both wild-type or Kindlin-2 knockout PCCs (Figure S8), which is consistent with the results showing that Kindlin-2 does not regulate c-Myc 3'UTR-mediated mRNA translation (Figure S5). Collectively, these findings support the hypothesis that Kindlin-2 helps DDX3X bind to the 5' UTR of c-Myc mRNA and facilitates c-Myc protein translation. The next question we addressed is whether Kindlin-2 directly or indirectly binds to c-Myc mRNA to regulate its translation. We performed RIP followed by RT-PCR assay. The results showed that c-Myc mRNA was present in the Kindlin-2 immunoprecipitants **(**Figure 5L). To verify this, we performed RNA pull-down experiments using biotinylated c-Myc 5' UTR or 3' UTR mRNA and found that c-Myc 5'UTR, but not 3' UTR mRNA, was associated with Kindlin-2 **(**Figure 5M-N). To further investigate whether Kindlin-2 directly interacts with the c-Myc 5' UTR, we utilized *in vitro* purified GST-Kindlin-2 to do RNA pull-down assay. The results revealed that GST-Kindlin-2 alone couldn't interact with c-Myc 5' UTR, however, the addition of GST-DDX3X significantly enhanced the association of Kindlin-2 with c-Myc 5'UTR **(**Figure 5O). Collectively, these results suggest that Kindlin-2 regulates c-Myc translation, at least in part, through its interaction with DDX3X and thereby control 5'UTR-mediated translation **(**Figure 5). Collectively, these results indicated that Kindlin-2 regulates c-Myc translation, at least in part, via 5'UTR-mediated translation control, and this event may be mediated by interaction with DDX3X.

### Kindlin-2 deletion reduces glycolysis via regulation of c-Myc downstream signaling

**Figure 6:**
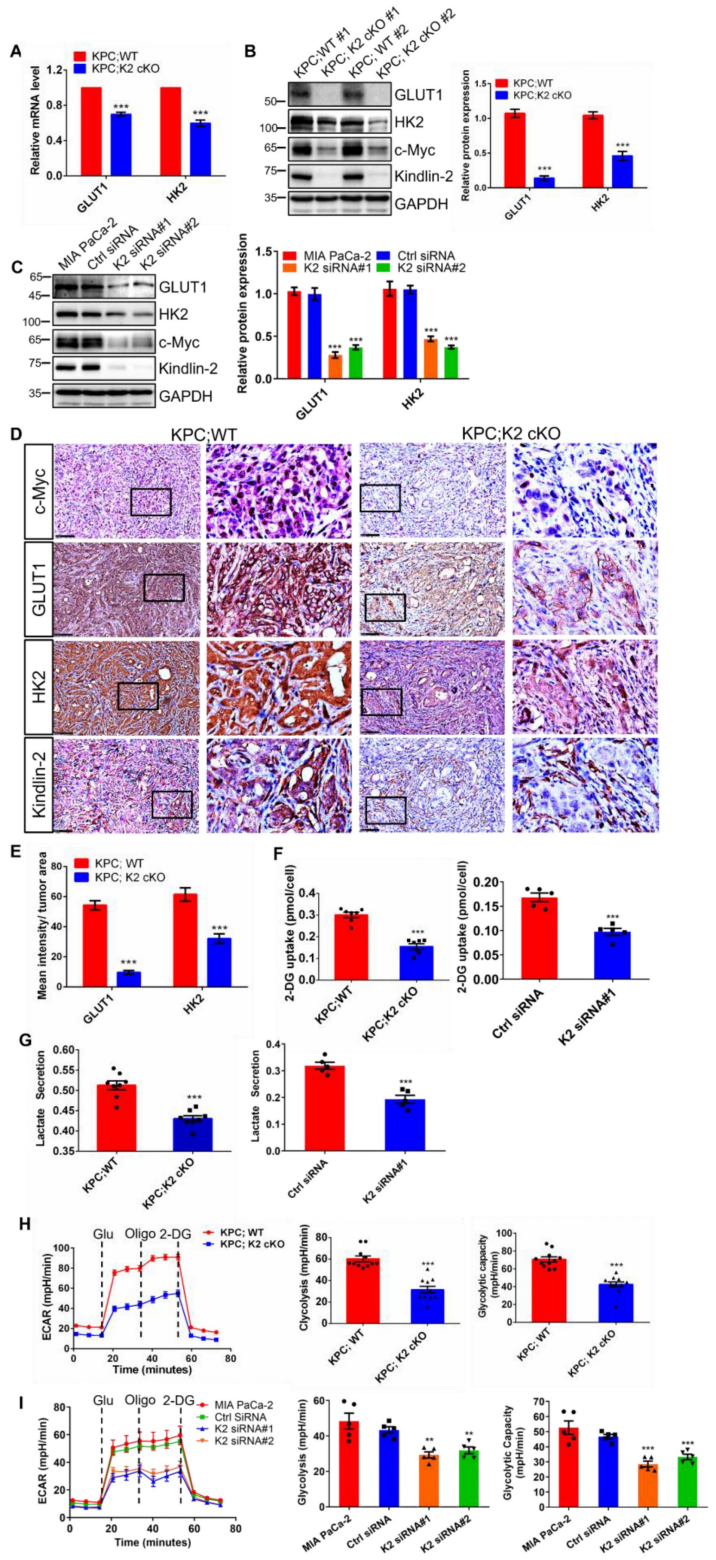
** Kindlin-2 deletion reduces c-Myc downstream targets GLUT1 and HK2 expression and inhibits glycolysis in pancreatic cancer cells. (A)** qPCR analysis of Glucose transporter 1 (*GLUT1*) and hexokinase 2 (*HK2*) mRNA expression in PCCs. ***P < 0.001 vs. KPC;WT. *n* = 4 independent experiments.** (B)** Immunoblotting analysis of GLUT1 and HK2 protein levels in PCCs (left panel). Quantification data were shown in the right panel. ***P < 0.001 vs. KPC;WT. *n* = 3 independent experiments.** (C)** Immunoblotting analysis of GLUT1 and HK2 protein levels in control (Ctrl siRNA) and Kindlin-2 knockdown (K2 siRNA#1 and K2 siRNA#2) MIA PaCa-2 cells. Quantification analysis was shown in the right panel. *** *P* < 0.001 vs. MIA PaCa-2. *n* = 3 independent experiments.** (D)** Representative images of pancreatic tumor sections stained with antibodies as indicated in the figure. Scale bar: 100 µm. **(E)** Quantification of staining intensity of GLUT1 and HK2 levels in pancreatic tumor sections. ***P < 0.001 vs. KPC;WT. *n* = 4 mice. For each mouse, the quantification was performed from at least ten images. **(F)** The relative glucose uptake was measured in PCCs or Human MIA PaCa-2 cells. ***P < 0.001 vs. KPC;WT, *n* = 7 independent experiments for PCCs (left panel); ***P < 0.001 vs. Ctrl siRNA, *n* = 5 for MIA PaCa-2 cells (right panel).** (G)** Lactate production in PCCs or Human MIA PaCa-2 cells. Levels of lactate in the culture medium were measured and normalized to the cell number. ***P < 0.001 vs. KPC;WT, *n* = 8 independent experiments for PCCs (left panel); ***P < 0.001 vs. Ctrl siRNA, *n* = 5 for MIA PaCa-2 cells (right panel).** (H and I)** Glycolysis flux was examined by measuring the extracellular acidification rate (ECAR) using the Seahorse analyzer in PCCs **(H)** or in Human MIA PaCa-2 cells **(I)**. Glucose (10 mM), ATP synthase inhibitor oligomycin (1 µM), and glycolysis inhibitor 2-Deoxy-D-glucose (2-DG, 50 mM) were added to the cells at the indicated time points. The values of glycolysis and glycolytic capacity were calculated by the Seahorse XFe96 software and shown in the right panel. ***P < 0.001 vs. KPC;WT, *n* = 11 independent experiments for PCCs **(H)**; ***P < 0.001, **P < 0.01 vs. Ctrl siRNA, *n* = 5 for MIA PaCa-2 cells **(I)**.

c-Myc is essential for promoting glucose metabolic reprogramming in various types of cancers 58. In particular, c-Myc functions as a key factor in reprogramming glucose metabolism by directly controlling glucose transporter (GLUT1) and rate-limiting glycolytic enzymes Hexokinase II (HK2) transcription in PDAC 59. Given our aforementioned data that revealed how Kindlin-2 influences c-Myc protein level (Figure 4) and metabolic gene transcripts (Figure 3), we examined whether Kindlin-2 could affect c-Myc downstream targeted genes including GLUT1 and HK2 to promote PDAC progression. Indeed, both mRNA and protein levels of GLUT1 and HK2 were dramatically reduced in Kindlin-2 knockout PCCs compared to control cells (Figure 6A-B). In line with these findings, a significant reduction of c-Myc, GLUT1, and HK2 expression was observed in Kindlin-2 knockdown human MIA PaCa-2 cells and in the Kindlin-2-deficient PDAC tumor section (Figure 6C-E). Since GLUT1 and HK2 are critically involved in glycolysis, we reasoned that Kindlin-2 might affect glycolysis in PDAC. To test this hypothesis, we measured glucose uptake in wild-type and Kindlin-2 knockout PCCs. The analysis revealed that the uptake of the glucose analog, 2-deoxyglucose (2-DG), was significantly diminished in both Kindlin-2 knockout PCCs and Kindlin-2 knockdown MIA PaCa-2 cells (Figure 6F). Concomitant to glucose uptake analysis, Kindlin-2-deficient PCCs and MIA PaCa-2 cells displayed remarkable decreases in lactate production (Figure 6G). In addition, the extracellular acidification rates (ECARs) were evaluated. The results showed that glycolysis and glycolytic capacity were markedly reduced by depletion of Kindlin-2 (Figure 6H-I), further supporting a role for Kindlin-2 in regulating tumor cell glycolytic metabolism reprogramming to promote PDAC progression.

**Figure 7:**
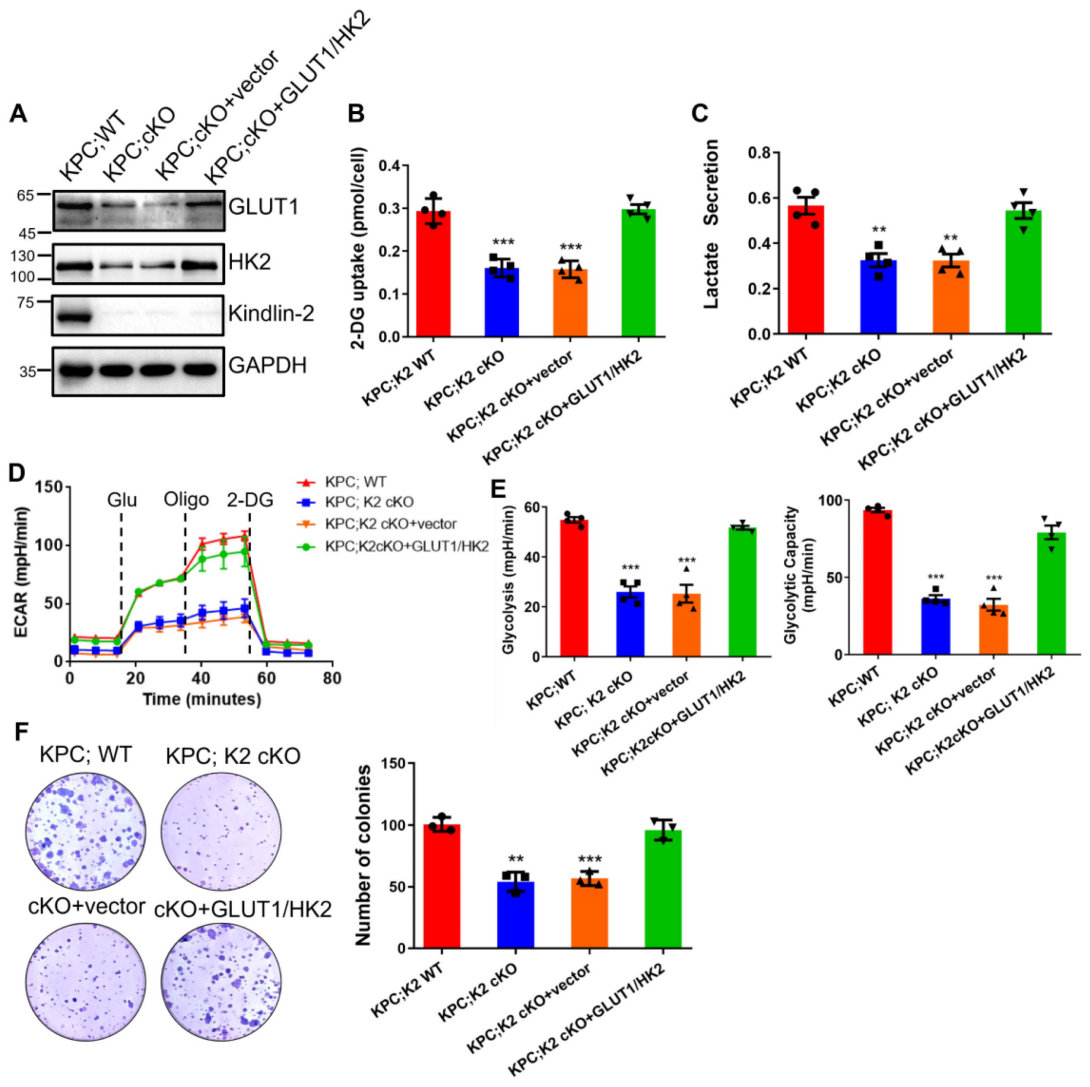
** Overexpression of GLUT1/HK2 rescues Kindlin-2-deficency-induced inhibition of glycolysis and pancreatic cancer cell proliferation.** Mouse primary PCCs isolated from KPC;K2 WT or KPC;K2 cKO mice were infected with lentiviral vectors encoding full-length GLUT1 and HK2 (GLUT1/HK2) or empty vector pLVX-IRES-Hyg.** (A)** Immunoblotting analysis of GLUT1 and HK2 protein levels in PCCs (different groups as specified in the figure). **(B)** The relative glucose uptake was measured in PCCs (different groups as specified in the figure). ***P < 0.001 vs. KPC;WT. *n* = 4 independent experiments.** (C)** Lactate production in PCCs as specified in the figure. Levels of lactate in the culture medium were measured and normalized to the cell number. **P < 0.01 vs. KPC;WT. *n* = 4 independent experiments. **(D)** Glycolysis flux was examined by measuring the extracellular acidification rate (ECAR) using the Seahorse analyzer. Glucose (10 mM), ATP synthase inhibitor oligomycin (1 µM), and glycolysis inhibitor 2-DG (50 mM) were added to the cells as indicated in the figure at the indicated time points. **(E)** The values of glycolysis and glycolytic capacity were calculated by the Seahorse XFe96 software. ***P < 0.001 vs. KPC;WT. *n* = 4 independent experiments.** (F)** Overexpression of GLUT1 and HK2 in Kindlin-2-deficient PCCs led to a significant increase in anchorage-dependent colony-forming ability. Representative images (left panel) and quantification analysis (right panel) were shown. ***P < 0.001, **P < 0.01 vs. KPC;WT. *n* = 3 independent experiments.

To further test the importance of glycolysis in Kindlin-2-regulated PDAC progression, we overexpressed GLUT1 and HK2 in Kindlin-2 knockout PCCs and found the forced increase of GLUT1 and HK2 expression significantly restored Kindlin-2-deficiency-induced inhibition of glycolysis and PCCs proliferation (Figure 7A-F), suggesting that Kindlin-2-deficiency-induced inhibition of PDAC progression is indeed mediated by, at least in part, suppression of glycolysis.

### c-Myc expression level is crucial for Kindlin-2-mediated regulation of glycolysis and pancreatic cancer cell growth

**Figure 8:**
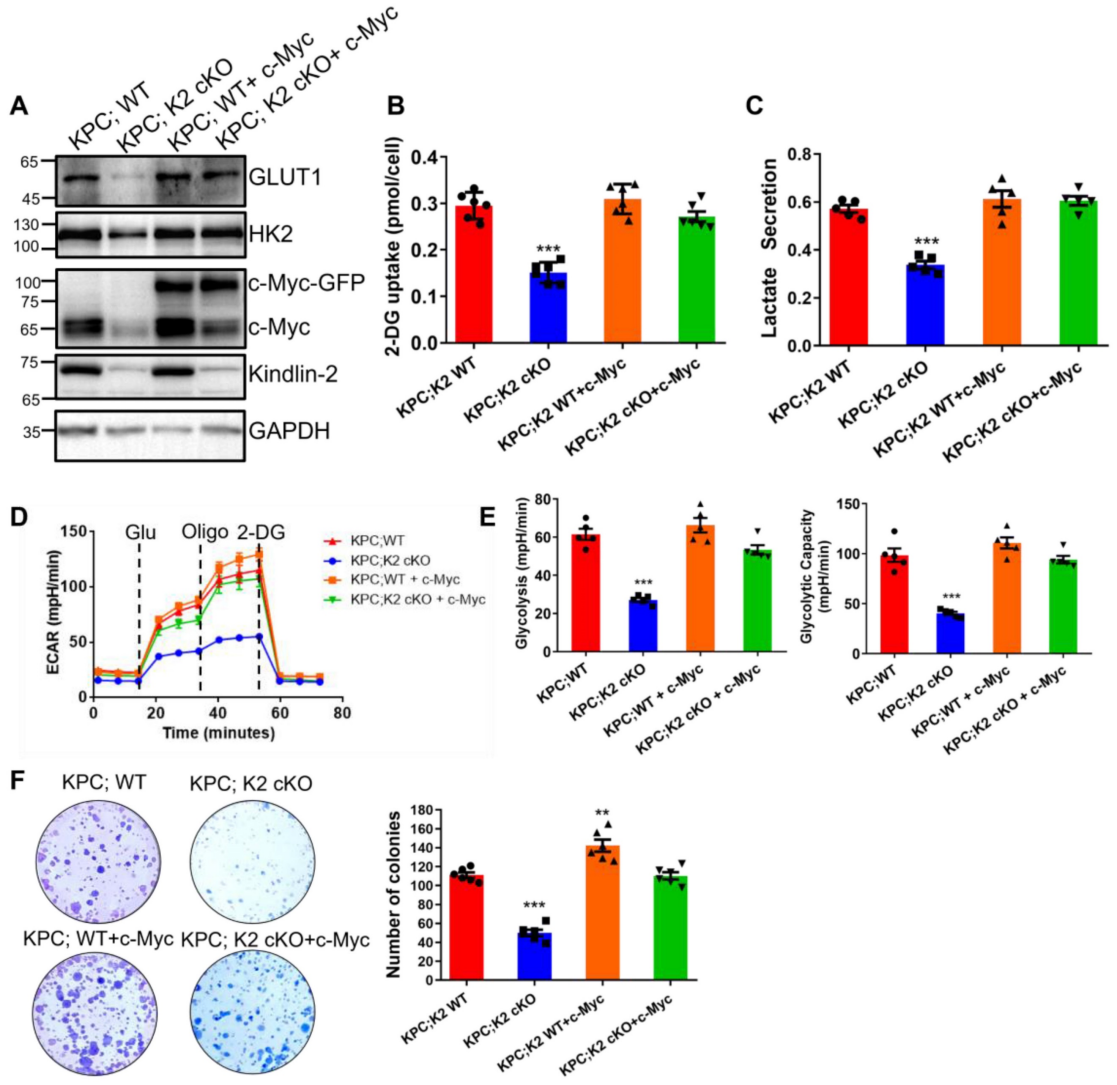
** c-Myc expression is crucial for Kindlin-2-mediated regulation of glycolysis and pancreatic cancer cell proliferation.** Mouse primary PCCs isolated from KPC;K2 WT or KPC;K2 cKO mice were infected with lentiviral vectors encoding full-length c-Myc (c-Myc-GFP) or empty vector GFP-tagged pLVX-IRES-Hyg (GFP).** (A)** Immunoblotting analysis of GLUT1 and HK2 protein levels in PCCs as specified in the figure. **(B)** The relative glucose uptake was measured in PCCs as specified in the figure. ***P < 0.001 vs. KPC;WT. *n* = 6 independent experiments.** (C)** Lactate production in PCCs as specified in the figure. Levels of lactate in the culture medium were measured and normalized to the cell number. ***P < 0.001 vs. KPC;WT. *n* = 5 independent experiments. **(D)** Glycolysis flux was examined by measuring the extracellular acidification rate (ECAR) using the Seahorse analyzer. Glucose (10 mM), ATP synthase inhibitor oligomycin (1 µM), and glycolysis inhibitor 2-DG (50 mM) were added to the cells as indicated in the figure at the indicated time points. **(E)**The values of glycolysis and glycolytic capacity were calculated by the Seahorse XFe96 software. ***P < 0.001 vs. KPC;WT. *n* = 5 independent experiments.** (F)** Overexpression of c-Myc in Kindlin-2-deficient PCCs led to a significant increase in anchorage-dependent colony-forming ability. Representative images (left panel) and quantification analysis (right panel) were shown. ***P < 0.001, **P < 0.01 vs. KPC;WT. *n* = 6 independent experiments.

To further characterize Kindlin-2-deficiency-induced inhibition of glycolysis and PDAC progression is specifically related to the down-regulation of c-Myc, we increased c-Myc expression in both wild-type and Kindlin-2 knockout PCCs by lentivirus transduction of GFP-tagged c-Myc (Figure 8A). Although overexpression of c-Myc in wild-type PCCs did not further enhance the expression of GLUT1, HK2 and overall glycolysis rate (Figure 8A-E), the forced increase of c-Myc expression in Kindlin-2 deficient PCCs significantly increased the protein expression of GLUT1 and HK2 (Figure 8A). Likewise, the reintroduction of c-Myc effectively restored the Kindlin-2 deficiency-induced inhibition of glucose uptake and lactate production (Figure 8B-C). Consistent with these findings, the re-expression of c-Myc reversed Kindlin-2 deficiency-induced reduction of glycolysis and glycolytic capacity (Figure 8D-E). Moreover, the PCCs cell proliferation defect, caused by the loss of Kindlin-2 was rescued by the overexpression of c-Myc (Figure 8F). Thus, these findings demonstrated the effects of Kindlin-2 on PDAC glucose metabolism and tumor progression are mediated through, at least in part, control of c-Myc expression level.

### Kindlin-2-DDX3X association is crucial for regulation of c-Myc expression and its downstream events

**Figure 9:**
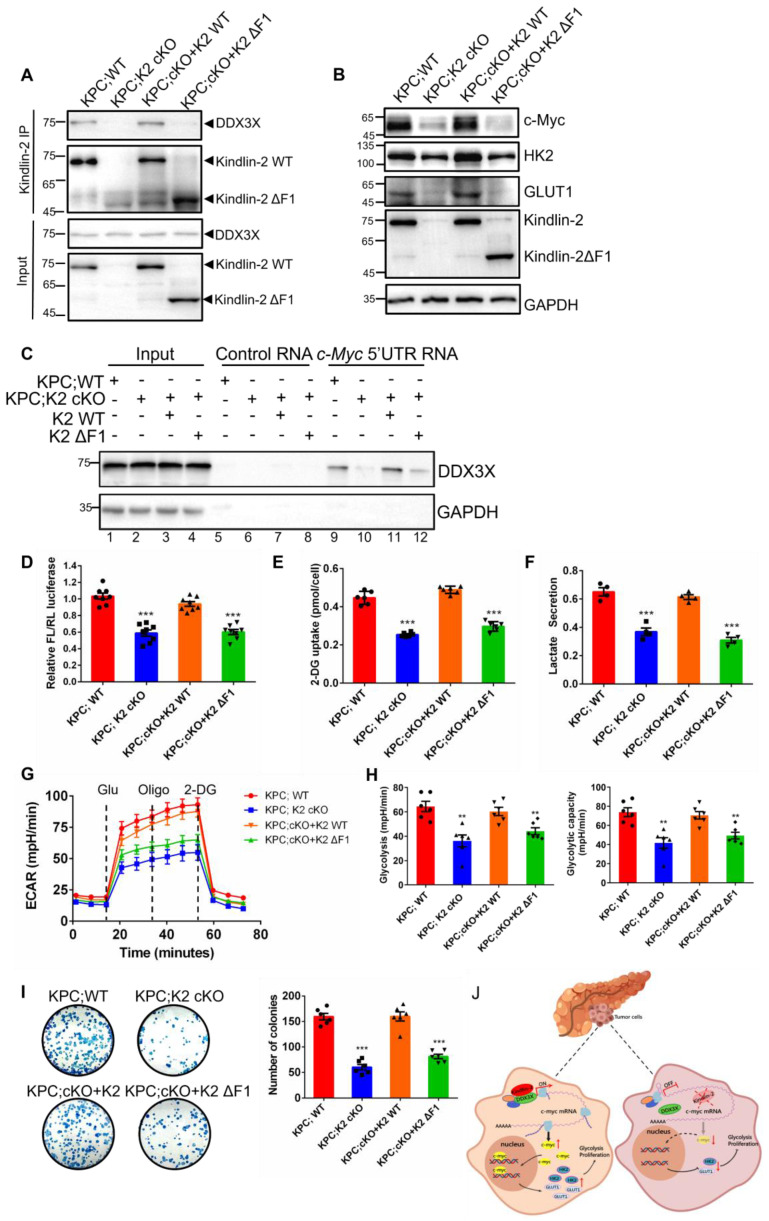
** Kindlin-2 association with DDX3X is crucial for the regulation of c-Myc translation and its downstream events.** Primary PCCs isolated from KPC;K2 WT or KPC;K2 cKO mice were infected with lentiviral vectors encoding wild-type Kindlin-2 (K2 WT) or F1-domain-deleted mutant of Kindlin-2 (K2 ΔF1) or empty vector pLVX-IRES-Hyg. **(A)** Cell lysates were immunoprecipitated with anti-Kindlin-2 antibody followed by immunoblotting with antibodies as indicated. **(B)** Immunoblotting analysis of c-Myc, GLUT1 and HK2 protein levels in PCCs as specified in the figure. **(C)** Pull-down assay using the biotinylated c-Myc 5'-UTR RNA with lysates of PCCs as specified in the figure, followed by immunoblotting analysis with antibodies as indicated. **(D)** Luciferase assay of c-Myc 5'-UTR-mediated translational activity in PCCs as specified in the figure. ***P < 0.001 vs. KPC;WT. *n* = 8 independent experiments.** (E)** The relative glucose uptake was measured in PCCs as specified in the figure. ***P < 0.001 vs. KPC;WT. *n* = 6 independent experiments.** (F)** Lactate production in PCCs as specified in the figure. Levels of lactate in the culture medium were measured and normalized to the cell number. ***P < 0.001 vs. KPC;WT. *n* = 4. **(G)** Glycolysis flux was examined by measuring the extracellular acidification rate (ECAR) using the Seahorse analyzer. Glucose (10 mM), ATP synthase inhibitor oligomycin (1 µM), and glycolysis inhibitor 2-DG (50 mM) were added to the cells as indicated in the figure at the indicated time points. **(H)** The values of glycolysis and glycolytic capacity were calculated by the Seahorse XFe96 software. **P < 0.01 vs. KPC;WT. *n* = 6 independent experiments.** (I)** Overexpression of K2 WT, but not K2 ΔF1, in Kindlin-2-deficient PCCs led to a significant increase in anchorage-dependent colony-forming ability. Representative images (left panel) and quantification analysis (right panel) were shown. ***P < 0.001 vs. KPC;WT. *n* = 6 independent experiments.** (J)** Schematic illustration of the mechanism of Kindlin-2 regulation of pancreatic cancer progression.

Our previous data have shown that the F1 subdomain of Kindlin-2 mediated Kindlin-2-DDX3X association (Figure 5H). To test whether Kindlin-2 and DDX3X interaction is important for the regulation c-Myc translation and its downstream signaling, we generated an F1 deletion mutant of Kindlin-2 (referred to as Kindlin-2 ΔF1 hereafter) and a full-length of Kindlin-2. We assessed whether Kindlin-2 ΔF1 mutant could disrupt the Kindlin-2-DDX3X association. As expected, full-length Kindlin-2 associated with DDX3X, whereas Kindlin-2 ΔF1 completely abolished the association with DDX3X, which indicates the F1 subdomain is required for Kindlin-2-DDX3X binding (Figure 9A). Next, we investigate whether Kindlin-2 ΔF1 could rescue Kindlin-2-deficiency-induced down-regulation of c-Myc and its subsequent downstream effects. As shown in Figure 9B, ectopic expression of full-length Kindlin-2 restored c-Myc protein expression, but Kindlin-2 ΔF1 failed to do so. In addition, Kindlin-2 ΔF1 mutant, unlike that of full-length Kindlin-2, could not enable DDX3X binding to c-Myc 5' UTR regions to facilitate c-Myc 5'-UTR mediated translation process (Figure 9C-D). Furthermore, re-expression of Kindlin-2 ΔF1 was unable to reverse Kindlin-2-deficiency induced c-Myc downstream dysfunctions including the reduction of GLUT1 and HK2 expression, the inhibition of glycolytic capacity and PCC proliferation inhibition (Figure 9B, E-I). Collectively, these results demonstrate that association with DDX3X is critical for Kindlin-2 to regulate c-Myc translation and its downstream glycolytic targets and to contribute to PDAC progression.

## Discussion

c-Myc is a proto-oncogene that can enhance various glycolytic genes expression to improve tumor cell glycolysis and augment tumor cell proliferation. c-Myc expression is tightly controlled in normal cells 60, and overexpression of c-Myc induces normal cell hyper-proliferation and transformation to promote tumor progression 61. c-Myc is aberrantly overexpressed in over 43.5% of human pancreatic cancers 62. Conditional ablation of c-Myc in a Kras^G12D^-induced pancreatic ductal adenocarcinoma mouse model significantly suppressed PDAC progression 63. This evidence strongly indicates that the overexpression or amplification of c-Myc is crucial for Kras^G12D^-driven pancreatic cancer progression.

c-Myc expression is controlled at multiple levels, including mRNA transcription, translation and c-Myc mRNA and protein stability. Recent studies indicated that c-Myc expression is modulated by translational control through both 5'UTR and 3'UTR regulation 52, 64-66. The interaction of RPBs (HuR, AUF1, TIAR) with c-Myc 3'UTR is crucial for the control of c-Myc translation 67, 68. Compared to 3'UTR modulation, the regulation of c-Myc 5'UTR in the control of c-Myc translation is largely unknown. Therefore, identifying key regulators of 5'UTR of c-Myc in the control of c-Myc translation is an important question in cancer biology. It has been previously reported that the 5'UTR of c-Myc mRNA contains a complex secondary structure that limits the efficiency of cap-dependent translation. DDX3X has been shown to regulate the translation of a subset of mRNAs with complex structured 5'UTR and/or high GC content through directly contacting transcript 5'UTRs 17, implying DDX3X possibly associates with c-Myc mRNA 5'UTR transcript to enhance its translation efficiency. This notion was confirmed by silencing DDX3X-induced inhibition of c-Myc translation (Figure S9).

Kindlin-2 is well documented to localize at focal adhesion to regulate integrin-mediated cell-ECM adhesion and signaling 27, 29, 69. In this study, we identified a novel function of Kindlin-2 in the regulation of c-Myc mRNA translation. Our findings indicated that in addition to focal adhesions, a fraction of Kindlin-2 could localize at ER regions and is associated with DDX3X. Our data also suggested that Kindlin-2 and DDX3X association is crucial for recruitment of DDX3X to the c-Myc 5'UTR. Specifically, Kindlin-2 deletion led to DDX3X dissociation from c-Myc 5'UTR although the loss of Kindlin-2 did not alter DDX3X expression level and sub-cellular location. Overexpression of full-length Kindlin-2 rescued DDX3X association with c-Myc mRNA and increased Kindlin-2-deficiency-induced down-regulation of c-Myc translation. However, the DDX3X-binding-mutant of Kindlin-2 (Kindlin-2 ΔF1) failed to do so.

While our results strongly suggest that Kindlin-2 promotes PDAC progression through up-regulation of c-Myc expression and glycolysis, our studies do not exclude the possibility that other Kindlin-2-mediated processes can also contribute to the progression of PDAC. For example, Kindlin-2 is known to regulate TGF-β signaling, cell-ECM adhesion, cytoskeleton assembly, and ECM deposition, which likely also contributes to PDAC progression 25, 27, 29, 34, 70. However, the fact that re-expression of GLUT1 or HK2 expression in Kindlin-2 knockout PCCs significantly restored Kindlin-2 deficiency-induced inhibition of PCCs proliferation (Figure 7), strongly suggests that the Kindlin-2-mediated regulation of glucose metabolism is critically involved in this process.

Increased level of Kindlin-2 was observed in human and mouse PDAC lesions (Figure 1B-C) and was associated with poor clinical outcomes (Figure 1D-F), suggesting that Kindlin-2 overexpression is likely clinically significant in PDAC progression. Interestingly, Zhan et al. also reported that there was a correlation between increased expression of Kindlin-2 and poor prognosis of PDAC patients 70. The findings by us and Zhan et al. beg an important question, namely whether reducing Kindlin-2 expression can inhibit PDAC progression *in vivo*. Answering this question is crucial for the development of novel therapeutic approaches that target the Kindlin-2 signaling pathway to alleviate PDAC progression. As described here, our evidence supports a role for Kindlin-2 in promoting PDAC progression* in vivo* and therefore targeting the Kindlin-2 signaling pathway may provide a promising therapeutic approach to alleviate PDAC progression.

How does Kindlin-2 promote PDAC progression? In this study, we propose a model to explain a mechanistic link between increased expression of Kindlin-2 and c-Myc, a key pro-oncogene that drives PDAC progression (Figure 9J). In this model, in normal pancreatic cells or Kindlin-2 knockout pancreatic tumor cells with a low level of Kindlin-2, c-Myc maintained a low basal translation level because the 5'UTR of c-Myc mRNA contains a complex secondary structure that limits the efficiency of cap-dependent translation. However, in pancreatic tumor cells with a high level of Kindlin-2, Kindlin-2 associated with DDX3X and recruited more DDX3X to the 5'UTR of c-Myc complex structure, thereby promoting translation of c-Myc mRNA, further increasing c-Myc expression to promote tumor cell proliferation (Figure 9J). Although our studies have revealed a crucial role for the Kindlin-2 association with DDX3X in the regulation of c-Myc translation and PDAC progression, how Kindlin-2 affects DDX3X interaction with c-Myc mRNA remains unknown. Potentially, Kindlin-2's association with DDX3X may influence DDX3X's protein conformation. Alternatively, the Kindlin-2 association may affect the interaction of DDX3X with other translation initiation factors that are involved in c-Myc mRNA translation. Future studies are required to investigate these possibilities.

In summary, we have demonstrated a novel role for Kindlin-2 in the regulation of c-Myc translation and PDAC progression. Furthermore, we provide evidence suggesting that Kindlin-2 functions in this process through association with DDX3X, which facilitates DDX3X interaction with c-Myc mRNA 5'UTR and thereby promotes c-Myc translation and consequently tumor cell glycolysis and PDAC progression. Given the important role of the Kindlin-2-DDX3X-c-Myc signaling axis in PDAC progression, inhibition of this signaling axis may provide a promising therapeutic approach to alleviate PDAC progression.

## Materials and Methods

### Animal studies

*Kindlin-2^fl/fl^* transgenic mice were generated as previously described 36. *Kras^G12D/+^*; *Trp53^fl/fl^*; *Pdx1-Cre* (*KPC*) mice were purchased from the Jackson Laboratory. *Kindlin-2^fl/fl^* mice were crossed with *KPC* mice to generate *Kindlin-2^fl/+^*; *Kras^G12D/+^*; *Trp53^fl/fl^ and Kindlin-2^fl/fl^*; *Trp53^fl/fl^*;* Pdx1-Cre* mice. Intercrossing of their progeny results in the generation of *Kindlin-2^fl/fl^; Kras^G12D/+^*; *Trp53^fl/fl^*; *Pdx1-Cre* (KPC; K2 cKO) and *Kindlin-2^+/+^; Kras^G12D/+^*; *Trp53^fl/fl^*; *Pdx1-Cre* (KPC;K2 WT) littermates. Tail genotyping was performed by routine PCR protocol.

Tail genotyping was performed by routine PCR protocol. PCR primers used for analyzing were listed: (1) Kindlin-2: 5'-TGTGTTTCAAAGGTACTGGTCA-3'; 5'-ACAATGGTGCTTTGCCTACA-3'. (2) Cre: 5'-CCTGGACTACATCTTGAGTT GC-3'; 5'-AGGCAAATTTTGGTGTACGG-3'. (3) Kras: 5'-GCAGGTCGAGGGACCTAATA-3'; 5'-CTGCATAGTACGCTATACCCTGT-3'. (4) p53: 5'-GGTTAAACCCAGCTTGACCA-3'; 5'-GGAGGCAGAGACAGTTGGAG-3'.

All animal experiments were performed in accordance with the guidelines and regulations and approved by the Institutional Animal Care and Use Committee at the Southern University of Science and Technology of China.

### Cell culture

The HEK 293T cells and MIA PaCa-2 cells were purchased from American Type Culture Collection (ATCC). The cell lines were authenticated and performed mycoplasma contamination testing at the beginning of this study. HEK 293T cell lines were routinely cultured in Dulbecco's Modified Eagle's Medium (DMEM) /High glucose medium (Invitrogen, 11995-500) containing 10% FBS (Invitrogen, 10099-141) and 100 U/mL penicillin and 100 μg/mL streptomycin (Invitrogen, 15140-122). MIA PaCa-2 cell lines were cultured in Dulbecco's Modified Eagle's Medium (DMEM) /High glucose medium (Invitrogen, 11995-500) containing 10% FBS (Invitrogen, 10099-141), 2.5% Dark horse serum (Gibco, 26050088), and 100 U/mL penicillin and 100 μg/mL streptomycin (Invitrogen, 15140-122). Primary murine PDAC cells were isolated from 9-week-old KPC mice. Pancreatic tumor tissues were dissected and minced finely with sterile razor blades, digested with 1 mg/mL collagenase IV, 0.125 mg/mL dispase, 0.1% soybean trypsin inhibitor and 50 U/mL Dnase for 1 h at 37 ℃, and then incubated with pre-warmed 0.25% trypsin at 37 ℃ for 10 min, strained through a 100 μm cell strainer, re-suspended in 10 mL DMEM/High glucose medium with 10% FBS and seeded on 10 cm cell culture dish. Cells were incubated at 37 ℃ in a humidified atmosphere of 5% CO_2_.

### Immunohistochemical (IHC) staining of paraffin-embedded tissue slides

Paraffin-embedded human pancreatic cancer tissue microarrays (TMA) in Figure 1B were purchased from Shanghai Outdo Biotech (OD-CT-DgPan01-006). The correlation between Kindlin-2 expression and gender, age, TNM stage or number of lymph nodes was analyzed in human TMA samples in which the gender, age, TNM stage or number of lymph nodes was available.

Paraffin-embedded mouse pancreatic tumor tissues were prepared as described before 32. In brief, pancreatic tumor tissues were sectioned at a thickness of 3.5 μm and performed with Hematoxylin and eosin (H&E) staining according to the manufacturer's instructions. For histological and histopathological analyses, normal pancreas, early PanIN (ADM and PanIN1), late PanIN (PanIN2 and PanIN3) and PDAC were classified according to the standard described previously 71. At least three independent tumor sections in each group were analyzed. Masson's trichrome staining was performed with the Modified Masson's Trichrome Stain Kit (Cat#G1348, Solarbio Life Sciences, Beijing, China) according to the manufacturer's instructions.

Immunohistochemistry staining was carried out with the MaxVisionTM HRP-Polymer anti-Mouse/Rabbit IHC kit (Maxim, Fuzhou, China) as described previously 72. The following primary antibodies were used: Antibodies against Cytokeratin19 (CK19, Abcam, ab52625, 1:10000), F4/80 (CST, 70076S, 1:1000), CD3 (CST, 99940S, 1:300), Collagen I (Abcam, ab260043, 1:1000), α-smooth muscle actin (α-SMA, Sigma, A2547, 1:1000), Kindlin-2 (clone 3A3, Millipore, MAB2617, 1:5000), c-Myc (Abcam, ab32072, 1:100), Ki-67 (Maxim, MAB-0672). Sections were visualized using DAB and counterstained with hematoxylin. Sections of three individual pancreas tissues in each group were taken for analysis. Percentages of Ki-67^+^, F4/80^+^, CD3^+^ cells and CK19^+^, α-SMA^+^, and Collagen I^+^ areas were calculated using Image J software in at least ten randomly selected fields per section under 10 × objective.

### Lentiviral infection

Plasmid psPAX2, pMD2.G, pLVX-Flag-Hyg and pLVX-GFP-Hyg were obtained from Addgene. To generate wild-type Kindlin-2, Kindlin-2-ΔF1 or c-Myc overexpression cell lines, pLVX-K2-Hyg, pLVX-K2ΔF1-Hyg or pLVX-GFP-c-Myc-Hyg were co-transfected with psPAX2 and pMD2.G into HEK 293T cells. The lentivirus were collected from the HEK293T culture media on the third day after transfection, then filtered (pore size 45 µm) and concentrated by ultracentrifugation (50,000 × g, 2 h). Isolated primary murine PDAC cells were cultured in 6-well plate until 50% confluence and then replaced with fresh media containing lentivirus at a multiplicity of infection (MOI) of 100 mixed with 8 μg/mL polybrene. The infectious media were replaced with fresh complete media after 16 h, and then cultured for 48 h. The viral infection efficiency was confirmed by immunoblotting.

### Immunofluorescence staining

Isolated primary murine PDAC cells and MIA PaCa-2 cells were seeded on fibronectin-coated 20 mm diameter cover glasses in 12-well plates (1×10^5^ cells/well) and cultured in 37 ℃ for 24 h. Cells were then fixed with 4% PFA for 30 min, permeabilized with 0.1% Triton X-100 in 1× PBS for 10 min, blocked with 5% BSA for 30 min and incubated with anti-Kindlin-2 (clone 3A3, Millipore, MAB2617, 1:5000), anti-DDX3X (Proteintech, 11115-1-AP, 1:1000), anti-c-Myc (Abcam, ab32072, 1:100) antibodies overnight at 4 ℃. Cells were then washed with 1×PBS three times and incubated with secondary antibodies (Alex fluor, 1:500) for 30 min at room temperature. Cells were mounted with ProLong^TM^ Gold antifade reagent with DAPI (Invitrogen, P36941) and visualized by Zeiss LSM980 with an Airyscan2 imaging system.

### Immunoblotting analysis

Whole-cell extracts were prepared with 1% sodium dodecyl sulfate (SDS) lysis buffer with freshly added 1% proteinase inhibitor cocktail (MCE, HY-K0010) and 1mM phenylmethylsulfonyl fluoride (PMSF, Sigma-Aldrich, 32998-6). Protein concentrations were measured using the Pierce^TM^ BCA Protein Assay Kit (Thermo fisher, 23227) and equal amounts of proteins were loaded on SDS-PAGE gels. Immunoblotting analysis was performed as described previously 32. The following primary antibodies were used with indicated dilution: Kindlin-2 (Proteintech, 11453-1-AP, 1:1000), DDX3X (Proteintech, 11115-1-AP, 1:1000), c-Myc (Cell signaling, 5605S, 1:1000), GAPDH (ABclonal, AC035, 1:5000), GFP (Transgen, HT801, 1:2000), Flag (Sigma, F1804, 1:3000), GST (Transgen, HT601, 1:2000) and MBP (Cell signaling, 2396S, 1:1000).

### Co-Immunoprecipitation

Co-immunoprecipitation (Co-IP) analysis was performed as described before 72. Briefly, cells were harvested and lysed in cell lysis buffer (Beyotime, P0013) supplemented with 1% proteinase inhibitor cocktail and 1 mM PMSF for 30 min at 4 ℃. The supernatants of cell lysates after centrifuging were pre-cleared with protein A/G Plus-Agarose (Santa Cruz, sc-2003) for 1 h at 4 ℃. Then, equal amounts of cell lysates (2 mg) were incubated with anti-Kindlin-2 antibody (clone 3A3, Millipore, MAB2617), anti-DDX3X antibody (Proteintech, 11115-1-AP), mouse anti-FLAG M2 (sigma, F1804), or control anti-mouse IgG antibody (Invitrogen), anti-rabbit IgG antibody (Invitrogen) overnight at 4 ℃, followed by incubating with Protein A/G Plus-Agarose for 2 h. Beads were rinsed three times in pre-cold 1× PBS buffer with 1 mM PMSF. Proteins were then eluted from beads by 1× SDS polyacrylamide gel electrophoresis loading buffer containing 10% β-mercaptoethanol and were subsequently subjected to immunoblotting.

### GST pull-down assay

For generation of GST-fusion proteins containing full-length or various mutants of Kindlin-2 and full-length DDX3X, cDNAs encoding Kindlin-2 or its fragments and DDX3X were cloned into pGEX-4T-1 vector. *Escherichia coli* strain BL21 was then transformed with the expression vectors. GST and GST-fusion proteins were purified from *E. coli* BL21 using Glutathione-Sepharose 4B matrix (GE Healthcare) according to the manufacturer's instructions. Purified proteins were resolved by SDS-PAGE to verify their size and purity. In pull-down assays, GST or GST-fusion proteins were bound to Glutathione-Sepharose, mixed with mouse-isolated primary pancreatic cancer cell lysates and incubated overnight at 4 ℃. Subsequently, the beads were washed three times with 1 ml of 1×PBS containing 0.2% Triton X-100. GST and GST fusion proteins bound to the beads were eluted and analyzed by immunoblotting.

### Cell proliferation assay

For cell proliferation assays, mouse-isolated primary pancreatic cancer cells were seeded as 2×10^4^ cells per well and cultured in complete growth medium. At the indicated time points, cells were collected and counted using a Countstar automated cell counter. For colony formation assays, mouse-isolated primary pancreatic cancer cells were seeded as 500 cells per well and cultured in complete growth medium. After 8 days, cell colonies were washed with 1×PBS, fixed with 4% paraformaldehyde (PFA) for 15 min and stained with Modified Giemsa Staining Solution (Beyotime, C0131) for 20 min. The colonies were then imaged and counted.

### RT-qPCR

Total RNA was isolated from cells using FastPure® Cell/Tissue Total RNA Isolation Kit V2 (Vazyme, RC112-01) according to the manufacturer's instructions. Then, the mRNA was reverse transcribed into cDNA with ReverTra Ace™ qPCR RT Master Mix (TOYOBO, 037400). cDNA samples were subjected to quantitative RT-PCR using LightCycler 480 SYBR® Green I Master (ROCHE, 04887352001) with an Applied Biosystems 7500 Real-Time PCR system. The level of GAPDH mRNA was used as an endogenous control for normalization. The primer sequences used for qRT-PCR were: (1) GAPDH: 5'-GTGAAGGTCGGAGTCAACGG-3' and 5'-TCCTGGAAGATGGTGATGGG-3'; HK2: 5'-CCCTGTGAAGATGTTGCCCACT-3' and 5'-CCTTCGCTTGCCATTACGCACG-3'; GLUT1: 5'-GCTTCTCCAACTGGACCTCAAAC-3' and 5'-ACGAGGAGCACCGTGAAGATGA-3'; c-Myc: 5'-TCGCTGCTGTCCTCCGAGTCC-3' and 5'-GGTTTGCCTCTTCTCCACAGAC; cyclin-D1: 5'-GCAGAAGGAGATTGTGCCATCC-3' and 5'-AGGAAGCGGTCCAGGTAGTTCA-3'.

### RNA sequencing

Isolated primary murine PDAC cells were extracted with TRIzol reagent (Invitrogen) according to the manufacturer's instructions. After verification of the RNA integrity by Agilent 2100 bioanalyzer, the extracted RNA was reverse-transcribed to create a cDNA library using the NEBNext Ultra™ RNA Library Prep Kit for subsequent sequencing. The clustering of the index-coded samples was performed on a cBot Cluster Generation System using TruSeq PE Cluster Kit v3-cBot-HS (Illumina) according to the manufacturer's instructions and paired-end (150 bp) sequencing was performed by Chi-Biotech (Shenzhen, China). Three biological repeats of each cell sample were processed. Gene set enrichment assay (GSEA) and Encyclopedia of Genes and Genomes (KEGG) pathway analysis was performed using the OmicShare tools, a free online platform for data analysis (https://www.omicshare.com/tools).

### Luciferase assay

The 5'- or 3'-untranslated region (UTR) of c-Myc was amplified and inserted into the upstream of the firefly luciferase coding region in pFL-SV40 (Addgene) to generate pFL-SV40-Myc. The control reporter pRL-SV40 was purchased from YouBio. The pFL-SV40-Myc and control reporter pRL-SV40 were transfected into mouse-isolated primary pancreatic cancer cells and cultured for 48 h. The luciferase activities in different groups were processed using Dual-Luciferase® Reporter Assay System (Promega) according to the manufacturer's instruction and quantified by a microplate reader (EnSpire).

### RNA immunoprecipitation (RIP) assay

RIP assay was performed using RNA Immunoprecipitation Kit (Geneseed, P0101, China) according to the manufacturer's instructions. In brief, mouse-isolated primary pancreatic cancer cells were lysed with RIP buffer and incubated with protein A/G beads pre-coated with anti-DDX3X antibody (Proteintech, 11115-1-AP) for 2 h at 4 ℃. Then, the mRNAs and proteins eluted from the beads were processed for qRT-PCR analysis.

### Polysome profiling assay

Cells were grown to ~80% confluence in 150 mm culture dishes and treated with 100 μg/mL cycloheximide at 37 °C for 5 min before the collection to halt the elongation. Cells were collected and lysed in lysis buffer [300 mM NaCl, 20 mM Tris-HCl (pH 7.4), 10 mM MgCl, 1% Triton X-100, 1 mM DTT, 0.5% sodium deoxycholate, protease inhibitor cocktail, phosphatase inhibitor] and incubate on ice for 15 min. Lysates were centrifuged at 13,000 g for 5 min and the supernatant was collected. Then, the supernatant was carefully layered onto the top of the 7% ~ 47% sucrose gradient. Gradients were then centrifuged at 35,000 g for 3 h at 4 °C and polysome-bound fractions were collected using a Peak Detector software with Foxy fractionator and UA-6 continuous UV detector. The RNA in each fraction was isolated with TRIzol and was subjected to reverse transcription and quantitative RT-PCR analysis.

### Biotin-labeled RNA pull-down assay

c-Myc 5'- or 3'-UTR RNA was synthesized from linearized DNA templates containing the T7 RNA promoter sequence followed by the c-Myc 5'- or 3'-UTR using T7 RiboMAX^TM^ Large Scale RNA Production Systems (Promega, P1300) according to the manufacturer's instructions. 50 pmol of c-Myc 5'- or 3'-UTR RNA was then biotinylated using Pierce RNA 3'-End Desthiobiotinylation Kit (Thermo, 20163) and captured with 50 μL of streptavidin magnetic beads (Provided in RBP Enrichment Module, Thermo, 20164Y). Subsequently, the beads were washed three times with washing buffer (Provided in RBP Enrichment Module, Thermo, 20164Y). RNA-binding proteins were eluted from the beads using Biotin Elution Buffer and analyzed by immunoblotting.

### Glycolysis stress test

Glycolysis stress test was performed using the Seahorse XF Glycolysis Stress Test Kit (Agilent, 103020-100) according to the manufacturer's instructions. Briefly, 5000 cells/well were seeded in Seahorse XF cell culture microplates with growth medium and incubate 24 h at 37 °C in a humidified incubator with 5% CO_2_. Wash and replace the culture medium with XF assay medium (Seahorse XF DMEM medium, pH 7.4, with 2 mM L-Glutamine), and incubate for an hour in a 37 °C non-CO_2_ incubator. The extracellular acidification rate (ECAR) was measured by XFe96 Analyzer in XF assay medium following sequential additions of glucose (10 mM), oligomycin (1 μM) and 2-DG (50 mM). Data were analyzed by XF Report Generators.

### Glucose uptake assay

Glucose uptake assay was performed using a Glucose uptake assay kit (Fluorometric, Abcam, ab136956) according to the instructions. Briefly, 1×10^5^ cells were seeded in 24-well plates and cultured in 37 ℃ with 5% CO_2_ for 24 h. Cells were then starved in serum free medium overnight to increase glucose uptake. Next day, cells were washed with 1×PBS three times and starved for glucose by incubating with Krebs-Ringer bicarbonate HEPES (KRBH) buffer containing 2% BSA for 40 min. 1 mM 2-deoxy-D-glucose (2-DG) was then added to the cells and incubated for additional 20 min. After that, cells were digested and measured for 2-DG uptake.

### Lactate assay

To measure lactate production, 1×10^5^ cells were seeded in a 24-well plate and cultured in 37 ℃ with 5% CO_2_ for 24 h. Cells were then cultured for additional 12 h in fresh complete medium and collected to measure lactate concentration with CheKine^TM^ Lactate Assay Kit (Abbkine, KTB1100) according to the manufacturer's instruction.

### Statistical analyses

All data represent as mean ± SEM. 2-tailed Student's *t* test was used to compare two groups of samples. One-way ANOVA was used for multiple comparisons. Survival analysis was carried out using the log-rank test. *P* values less than 0.05 were considered significant. Prism 7 (GraphPad) was used for statistical analysis.

## Supplementary Material

Supplementary figures.

## Abbreviations

immune-precipitation): PDAC (pancreatic ductal adenocarcinoma
