# Supplementary material for: Kindlin-2 enhances c-Myc translation through association with DDX3X to promote pancreatic ductal adenocarcinoma progression
Source: Theranostics. 2023 Jul 31;13(13):4333–55. doi: 10.7150/thno.85421 (PMC10465218; doi:10.7150/thno.85421)
Supplement: Supplementary file 1 — Supplementary figures. [file thnov13p4333s1.pdf]

Supplementary Material:

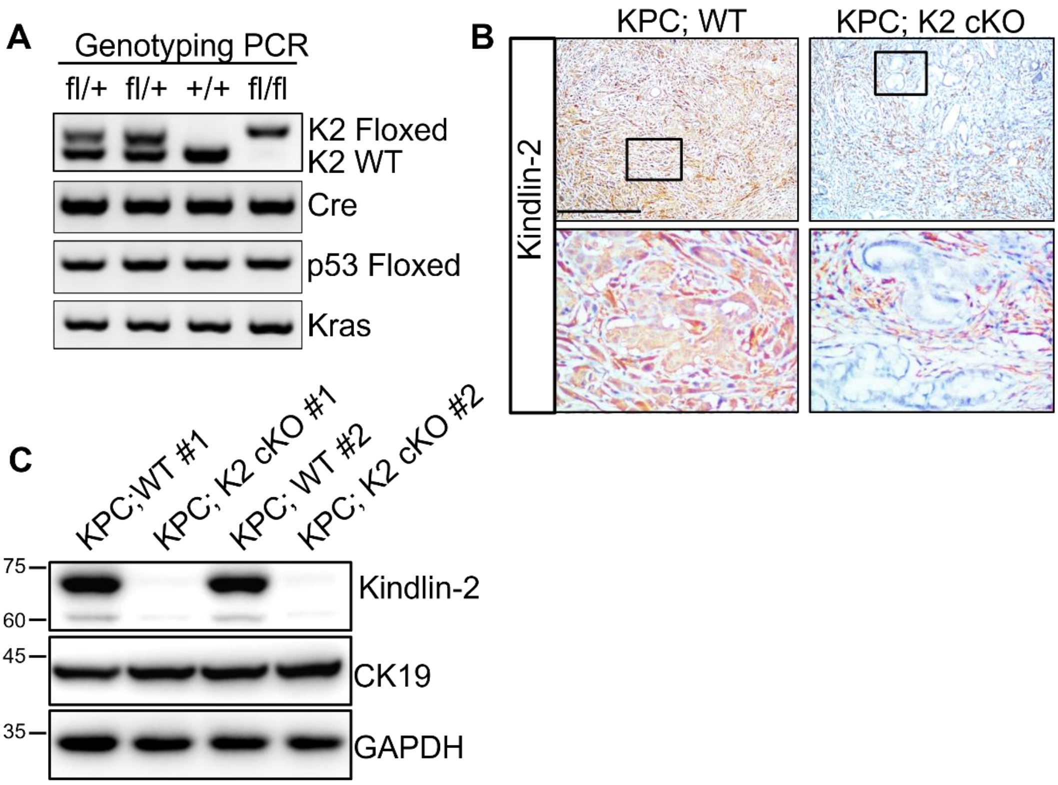

Supplementary Figure 1. Generation of KPC; Kindlin-2 cKO mice (KPC; K2 cKO). (A) Representative PCR analysis of extracted genomic DNA from tail clippings. PCR product bands of Kindlin-2 (K2) floxed (640 bp) and K2 WT (572 bp) were shown. p53 floxed, $Kras^{LSL\text{-}G12D}$ (Kras) and Cre PCR products were also indicated. **(B)** Representative images of pancreatic tumor tissues from KPC;WT and KPC;K2 cKO littermates at 9-week-age stained with anti-Kindlin-2 antibody. Scale bar: 100 μm. The lower panels of **(B)** showed higher magnification images of the areas outlined with black squares in the upper panels. **(C)** Immunoblotting analysis of Kindlin-2 expression in primary pancreatic cancer cells (PCCs) isolated from two paired KPC;WT and KPC;K2 cKO littermates. Cytokeratin 19 (CK19) was used as

a marker of pancreatic ductal adenocarcinomas.

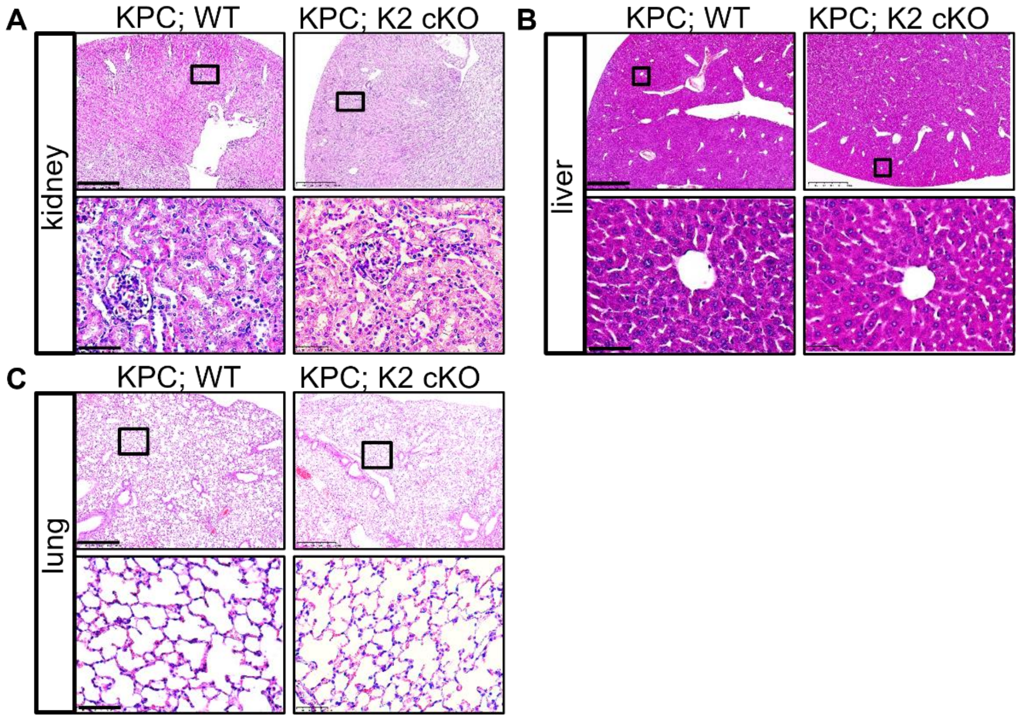

Supplementary Figure 2. KPC;K2 cKO mice display normal development of a set of key organs, such as kidney, liver, and lungs. (A-C) Representative histologic images of H&E staining in kidney **(A)**, liver **(B)** and lung sections **(C)** from KPC;WT and KPC;K2 cKO. Scale bar: 1 mm (upper panel); 50 μm (lower panel).

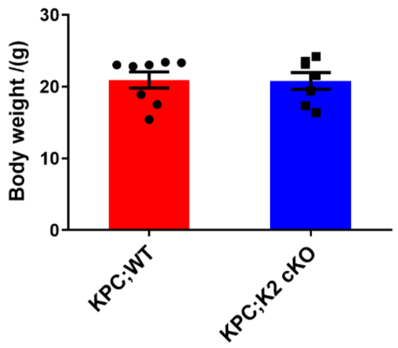

Supplementary Figure 3. Comparison of body weights between KPC;WT and KPC;K2 cKO mice at 7-week-age.

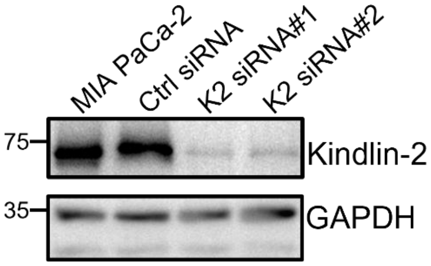

Supplementary Figure 4. Knockdown of Kindlin-2 in human pancreatic cancer cells MIA PaCa2. Immunoblotting analysis of Kindlin-2 protein expression in control (Ctrl siRNA) and Kindlin-2 knockdown (K2 siRNA#1 and K2 siRNA#2) MIA PaCa-2 cells.

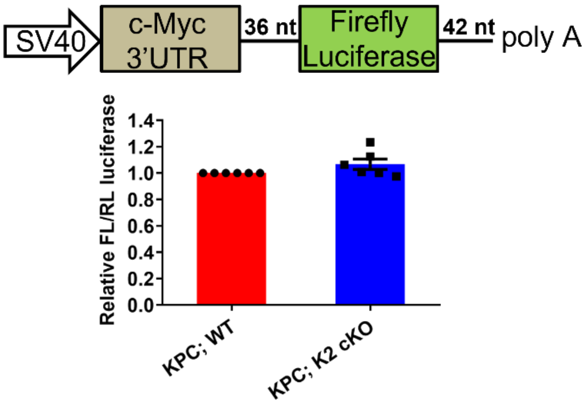

Supplementary Figure 5. Loss of Kindlin-2 does not alter c-Myc 3'UTR-mediated translation. Upper panel: schematic of c-Myc 3'UTR-mediated translation (*Firefly* luciferase as a reporter gene). Lower panel: luciferase assay of c-Myc 3'UTR-mediated translational activity in primary PCCs. *n* = 6 independent experiments. FL, *firefly*; RL, *Renilla*.

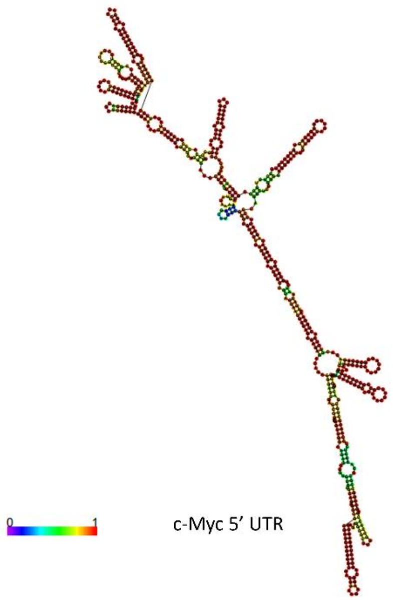

Supplementary Figure 6. Schematic representation of the 5’-UTR of c-Myc mRNAs.

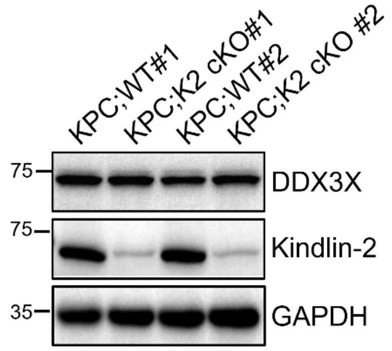

Supplementary Figure 7. Depletion of Kindlin-2 did not alter DDX3X protein expression. Immunoblotting analysis of DDX3X protein levels in PCCs isolated from two paired KPC;WT and KPC;K2 cKO littermates.

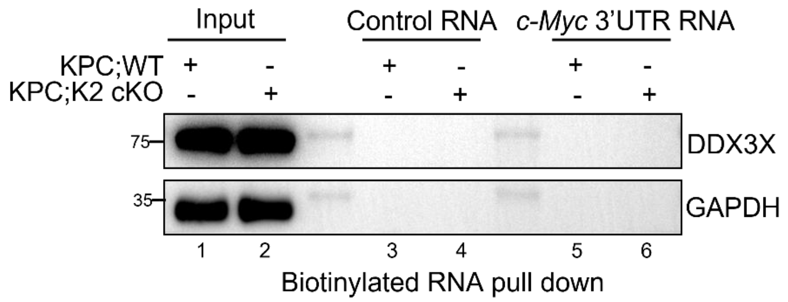

Supplementary Figure 8. DDX3X does not associate with c-Myc 3'UTR. Pull-down assay using the biotinylated c-Myc 3'-UTR RNA with cell lysates of mouse primary PCCs, followed by immunoblotting analysis with antibodies as indicated.

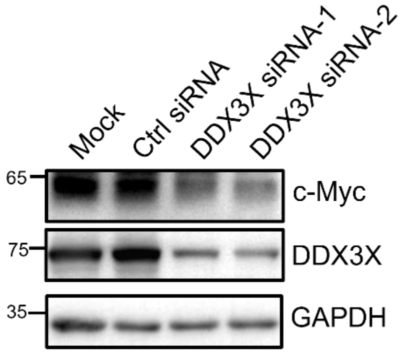

Supplementary Figure 9. Depletion of DDX3X reduced c-Myc expression. Knockdown of DDX3X in mouse primary pancreatic cancer cells (PCCs) using two independent siRNA, siRNA-1 and siRNA-2. The protein levels of c-Myc and DDX3X were evaluated by immunoblotting with antibodies as indicated. GAPDH was used as the loading control.
